# Effect of methyl­ene *versus* ethyl­ene linkers on structural properties of *tert*-butyl and mesityl bis­(imidazolium) bromide salts

**DOI:** 10.1107/S2056989022008003

**Published:** 2022-08-16

**Authors:** Emily S. Thompson, Elisa M. Olivas, Adrian Torres, Briana C. Arreaga, Hector L. Alarcon, Deandrea Dolberry, Jacob P. Brannon, S. Chantal E. Stieber

**Affiliations:** a Department of Chemistry & Biochemistry, California State Polytechnic University, Pomona, 3801 W. Temple Ave., Pomona, CA 91768, USA; Tulane University, USA

**Keywords:** crystal structure, bis­(imidazolium), bidentate NHC, N-heterocyclic carbene

## Abstract

The crystal structures of ligand precursor bis­(imidazolium) salts 1,1′-methyl­enebis(3-*tert*-butyl­imidazolium) dibromide monohydrate, C_15_H_26_N_4_^+^·2Br^−^·H_2_O or [^*t*Bu^NHC_2_Me][Br]_2_·H_2_O, 1,1′-(ethane-1,2-di­yl)bis­(3-*tert*-butyl­imidazolium) dibromide dihydrate, C_16_H_28_N_4_^+^·2Br^−^·2H_2_O or [^*t*Bu^NHC_2_Et][Br]_2_·2H_2_O, 1,1′-methyl­enebis[3-(2,4,6-tri­methyl­phen­yl)imidazolium] dibromide dihydrate, C_25_H_30_N_4_^2+^·2Br^−^·2H_2_O or [^Mes^NHC_2_Me][Br]_2_·2H_2_O, and 1,1′-(ethane-1,2-di­yl)bis­[3-(2,4,6-tri­methyl­phen­yl)imidazolium] dibromide tetra­hydrate, C_26_H_32_N_4_^2+^·2Br^−^·4H_2_O or [^Mes^NHC_2_Et][Br]_2_·4H_2_O, are reported. At 293 K, [^*t*Bu^NHC_2_Me][Br]_2_·H_2_O crystallizes in the *P*2_1_/*c* space group, while [^*t*Bu^NHC_2_Et][Br]_2_·2H_2_O crystallizes in the *P*2_1_/*n* space group at 100 K. At 112 K, [^Mes^NHC_2_Me][Br]_2_·2H_2_O crystallizes in the ortho­rhom­bic space group *Pccn* while [^Mes^NHC_2_Et][Br]_2_·4H_2_O crystallizes in the *P*2_1_/*c* space group at 100 K. Bond distances and angles within the imidazolium rings are generally comparable among the four structures. All four bis­(imidazolium) salts co-crystallize with one to four mol­ecules of water.

## Abstract 2

The crystal structures of ligand precursor bis­(imidaozolium) salts 1,1′-methyl­enebis(3-*tert*-butyl­imidazolium) dibromide monohydrate, C_15_H_26_N_4_^+^·2Br^−^·H_2_O or [^*t*Bu^NHC_2_Me][Br]_2_·H_2_O, 1,1′-(ethane-1,2-di­yl)bis­(3-*tert*-butyl­imidazolium) dibromide dihydrate, C_16_H_28_N_4_^+^·2Br^−^·2H_2_O or [^*t*Bu^NHC_2_Et][Br]_2_·2H_2_O, 1,1′-methyl­enebis[3-(2,4,6-tri­methyl­phen­yl)imidazolium] dibromide dihydrate, C_25_H_30_N_4_^2+^·2Br^−^·2H_2_O or [^Mes^NHC_2_Me][Br]_2_·2H_2_O, and 1,1′-1,1′-(ethane-1,2-di­yl)bis­[3-(2,4,6-tri­methyl­phen­yl)imidazolium] dibromide tetra­hydrate, C_26_H_32_N_4_^2+^·2Br^−^·4H_2_O or [^Mes^NHC_2_Et][Br]_2_·4H_2_O, are reported. At 293 K, [^*t*Bu^NHC_2_Me][Br]_2_·H_2_O crystallizes in the *P*2_1_/*c* space group, while [^*t*Bu^NHC_2_Et][Br]_2_·2H_2_O crystallizes in the *P*2_1_/*n* space group at 100 K. At 112 K, [^Mes^NHC_2_Me][Br]_2_·2H_2_O crystallizes in the ortho­rhom­bic space group Pccn while [^Mes^NHC_2_Et][Br]_2_·4H_2_O crystallizes in the *P*2_1_/*c* space group at 100 K.

## 1. Chemical context

Bis(imidazolium) salts are common precursors for the synthesis of bidentate *N*-heterocyclic carbene (NHC_2_) ligands, which can be used to stabilize a variety of metal complexes and catalysts. Bis(imidazolium) salts, [^*R*^NHC_2_ *R* ^1^][*X*]_2_ are relatively modular in that modifications can be relatively easily made to exterior groups attached to each NHC (*R*), the moiety linking the two NHC groups (*R* ^1^), and the counter-ion (*X*). One general synthetic approach for synthesizing bis(imidazolium) salts is where two equivalents of an alkyl or aryl imidazole are combined with one equivalent of an organic dihalide reagent and refluxed to afford the final product (Gardiner *et al.*, 1999 ▸). A simplified procedure for a variety of ligand salts using pressure tubes resulting in yields that were generally over 80% was also reported (Scherg *et al.*, 2006 ▸). Some reports have gone even further to minimize solvent in the synthesis of these ligand precursors, including a solvent-free synthesis (Cao *et al.*, 2011 ▸, 2012 ▸). This implies that the exterior *R* groups can easily be modified by changing the alkyl or aryl group on the starting imidazole. The linking group *R* ^1^ and counter-ion *X* can be modified by changing the organic dihalide reagent. In this fashion, a library of bis­(imidazolium) salts can be relatively easily synthesized from alkyl or aryl imidazoles, and some are also commercially available.

Some of the most widely reported bis­(imidazolium) salts are those with *tert*-butyl (^*t*^Bu) and mesityl (Mes) exterior *R* groups and methyl­ene (Me) or ethyl­ene (Et) linking *R* ^1^ groups. [^Mes^NHC_2_Et][Br]_2_ was even reported to be a stand-alone catalyst for the conversion of aryl­aldehydes to carb­oxy­lic acids in combination with water and K_2_CO_3_ in DMSO (Yang *et al.*, 2013 ▸). Methyl­ene linkers are quite commonly used for complexing to metals, and although examples with ethyl­ene linkers are fewer, comparative studies report that changing the linker affects catalysis. For example, shorter methyl­ene linkers (*R* ^1^) were reported to be more effective for hydro­silylation reactions with Rh^I^ complexes than ethyl­ene linkers (Riederer *et al.*, 2010 ▸).

The bidentate NHC ligand system is highly versatile for stabilizing a range of metals, some of which result in catalytically active systems. For example, [^*t*Bu^NHC_2_Me][Br]_2_ and [^*t*Bu^NHC_2_Et][Br]_2_ were used as precursors for synthesis of rhodium complexes (Leung *et al.*, 2006 ▸). [^*t*Bu^NHC_2_Et][Cl]_2_ was used for synthesis of aluminum, gallium, and indium complexes (Baker *et al.*, 2002 ▸). [^Mes^NHC_2_Et][Br]_2_ was reported for synthesizing rhenium complexes (Hock *et al.*, 2014 ▸; Hiltner *et al.*, 2010 ▸), palladium complexes *via* Pd(OAc)_2_-assisted deprotometalation (Wierenga *et al.*, 2019 ▸), palladium complexes *via* silver transmetallation (Sluijter *et al.*, 2013 ▸), and for palladium catalysts for Suzuki and Heck coupling reactions (Lee *et al.*, 2004 ▸). Other reported palladium complexes were active for dehalogenation of aryl halides (Viciu *et al.*, 2001 ▸).

Examples with first row transition metals are fewer, with nickel being the most commonly reported. Nickel carbonato complexes were synthesized with [^Mes^NHC_2_Me][Cl]_2_ and [^Mes^NHC_2_Et][Cl]_2_ ligand precursors (Guo *et al.*, 2013 ▸). Iron complexes for use in aryl Grignard-alkyl halide cross-coupling reactions were synthesized using various bis­(imidazolium) salts including [^*t*Bu^NHC_2_Me][Cl]_2_, [^*t*Bu^NHC_2_Me][Br]_2_, [^Mes^NHC_2_Me][Cl]_2_, [^Mes^NHC_2_Me][Br]_2_, and [^Mes^NHC_2_Et][Br]_2_ (Meyer *et al.*, 2011 ▸).

When used for stabilizing bimetallic systems, [^*t*Bu^NHC_2_Et][Cl]_2_ and [^Mes^NHC_2_Et][Cl]_2_ have been used as precursors for dipalladium complexes for Heck reactions (Li *et al.*, 2013 ▸; Yang *et al.*, 2012 ▸; Cao *et al.*, 2010 ▸), while [^*t*Bu^NHC_2_Et][Br]_2_ was a precursor for dimetallic Rh complexes (Wells *et al.*, 2008 ▸) and mixed-metal Rh/Pd (Zamora *et al.*, 2009 ▸) and Ir/Rh (Frey *et al.*, 2006 ▸). Similarly, [^Mes^NHC_2_Me][Br]_2_ and [^Mes^NHC_2_Et][Br]_2_ were used to synthesize bimetallic gold catalysts for cross-coupling and hydro­amination reactions (Baron *et al.*, 2018 ▸). 
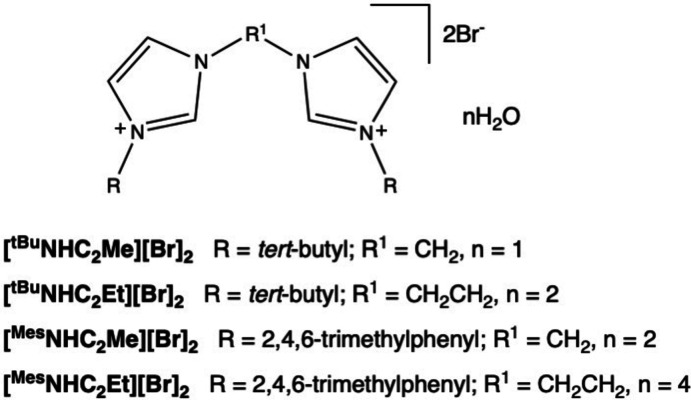


While bis­(imidazolium) salts are common ligand precursors, few have been structurally characterized (Rheingold, 2019 ▸). This work presents structural characterization and a comparison of supra­molecular features for methyl­ene- *versus* ethyl­ene-linked bis­(imidazolium) salts with *tert*-butyl and mesityl ancillary groups.

## 2. Structural commentary

**Figure 1:**
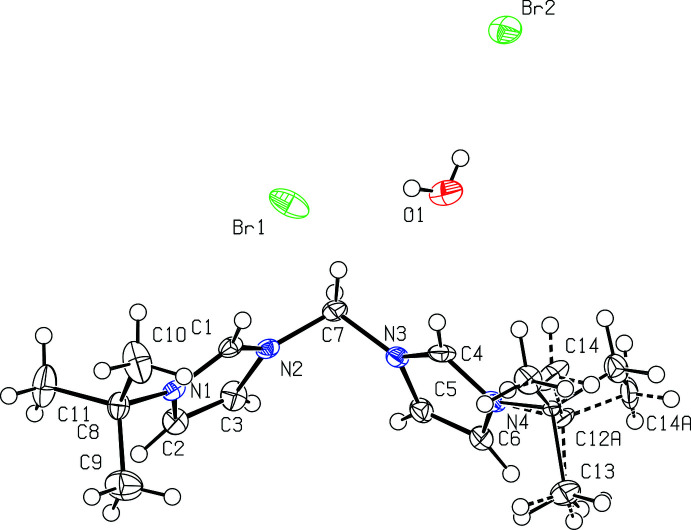
View of [^*t*Bu^NHC_2_Me][Br]_2_·H_2_O with 50% probability ellipsoids.

**Figure 2:**
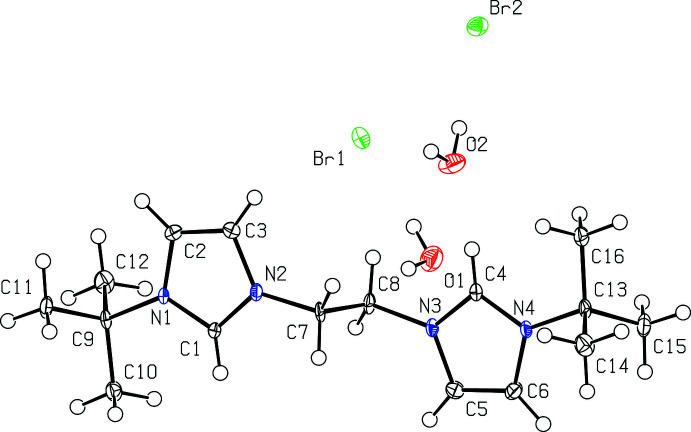
View of [^*t*Bu^NHC_2_Et][Br]_2_·2H_2_O with 50% probability ellipsoids.

All four bis­(imidazolium) salts were recrystallized from hot methanol and each compound co-crystallizes with one or more mol­ecules of water. Fig. 1 ▸ depicts [^*t*Bu^NHC_2_Me][Br]_2_·H_2_O while Fig. 2 ▸ depicts [^*t*Bu^NHC_2_Et][Br]_2_·2H_2_O.

Bond distances in the imidazolium rings of [^*t*Bu^NHC_2_Me][Br]_2_·H_2_O and [^*t*Bu^NHC_2_Et][Br]_2_·2H_2_O are mostly the same within experimental error, with backbone C2—C3 distances of 1.348 (4) and 1.349 (3) Å, respectively. The N—C distances are also mostly comparable with [^*t*Bu^NHC_2_Me][Br]_2_·H_2_O having an N1—C2 and an N2—C3 distance of 1.389 (3) Å and N1—C1 and N2—C1 distances both being 1.337 (3) Å, while [^*t*Bu^NHC_2_Et][Br]_2_·2H_2_O has an N1—C2 distance of 1.388 (3) Å, an N2—C3 distance of 1.384 (3) Å, an N1—C1 distance of 1.327 (3) Å and an N2—C1 distance of 1.331 (3) Å. For the linker, the N2—C7 distance is 1.463 (3) Å for [^*t*Bu^NHC_2_Me][Br]_2_·H_2_O and 1.468 (3) Å for [^*t*Bu^NHC_2_Et][Br]_2_·2H_2_O.

Bond angles in the imidazolium rings are also quite similar in [^*t*Bu^NHC_2_Me][Br]_2_·H_2_O and [^*t*Bu^NHC_2_Et][Br]_2_·2H_2_O. For [^*t*Bu^NHC_2_Me][Br]_2_·H_2_O, bond angles include C1—N1—C2 at 108.2 (2)°, N1—C2—C3 at 107.6 (2)°, C2—C3—N2 at 106.9 (2)°, C3—N2—C1 at 108.6 (2)°, and N2—C1—N1 at 108.7 (2)°. For [^*t*Bu^NHC_2_Et][Br]_2_·H_2_O, bond angles include C1—N1—C2 at 108.21 (19)°, N1—C2—C3 at 107.3 (2)°, C2—C3—N2 at 106.9 (2)°, C3—N2—C1 at 108.54 (19)°, and N2—C1—N1 at 109.02 (19)°.

**Figure 3:**
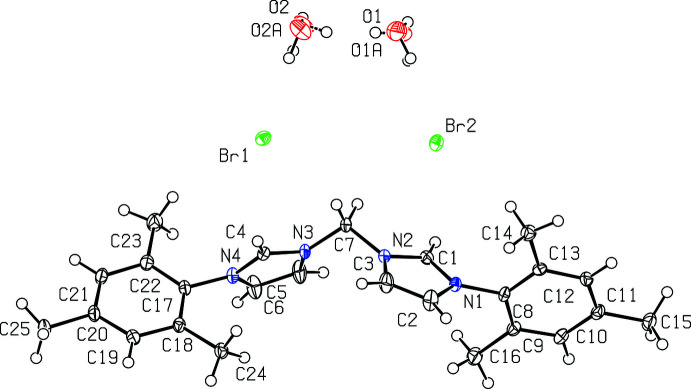
View of [^Mes^NHC_2_Me][Br]_2_·2H_2_O with 50% probability ellipsoids.

**Figure 4:**
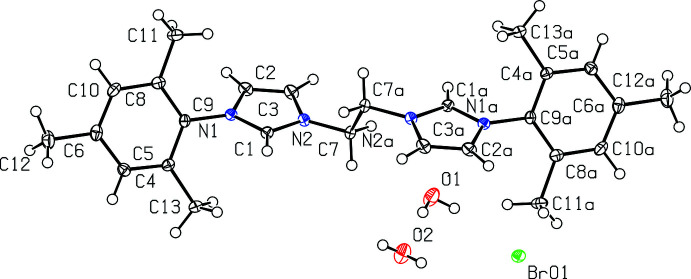
View of [^Mes^NHC_2_Et][Br]_2_·4H_2_O with 50% probability ellipsoids.

Fig. 3 ▸ depicts [^Mes^NHC_2_Me][Br]_2_·2H_2_O while Fig. 4 ▸ depicts [^Mes^NHC_2_Et][Br]_2_·4H_2_O. Notably, [^Mes^NHC_2_Et][Br]_2_·4H_2_O is the only compound of the four for which the asymmetric unit contains only half of the mol­ecule.

Bond distances in the imidazolium rings of [^Mes^NHC_2_Me][Br]_2_·2H_2_O and [^Mes^NHC_2_Et][Br]_2_·4H_2_O are mostly the same within experimental error, with backbone C2—C3 distances of 1.344 (3) and 1.3506 (19) Å, respectively. N—C distances are also mostly the same with [^Mes^NHC_2_Me][Br]_2_·2H_2_O having an N1—C2 distance of 1.387 (3) Å, an N2—C3 distance of 1.380 (3) Å, an N1—C1 distance of 1.326 (3) Å, and an N2—C1 distance of 1.341 (3) Å. Similarly, [^Mes^NHC_2_Et][Br]_2_·4H_2_O has an N1—C2 distance of 1.3872 (16) Å, an N2—C3 distance of 1.3841 (16) Å, an N1—C1 distance of 1.3322 (16) Å and an N2—C1 distance of 1.3314 (16) Å. For the linker, the N2—C7 distance is 1.457 (3) Å for [^Mes^NHC_2_Me][Br]_2_·2H_2_O and 1.4653 (16) Å for [^Mes^NHC_2_Et][Br]_2_·4H_2_O.

Bond angles in the imidazolium rings are also mostly the same for [^Mes^NHC_2_Me][Br]_2_·2H_2_O and [^Mes^NHC_2_Et][Br]_2_·4H_2_O. For [^Mes^NHC_2_Me][Br]_2_·2H_2_O, bond angles include C1—N1—C2 at 108.92 (17)°, N1—C2—C3 at 107.20 (19)°, C2—C3—N2 at 106.95 (19)°, C3—N2—C1 at 108.96 (17)°, and N2—C1—N1 at 107.96 (18)°. For [^Mes^NHC_2_Et][Br]_2_·4H_2_O, bond angles include C1—N1—C2 at 108.51 (11)°, N1—C2—C3 at 107.19 (11)°, C2—C3—N2 at 106.87 (11)°, C3—N2—C1 at 108.89 (11)°, and N2—C1—N1 at 108.54 (11)°. Overall, these data support that changing the linker *R* ^1^ group from methyl­ene to ethyl­ene does not significantly affect the imidazolium ring structures.

## 3. Supra­molecular features

**Figure 5:**
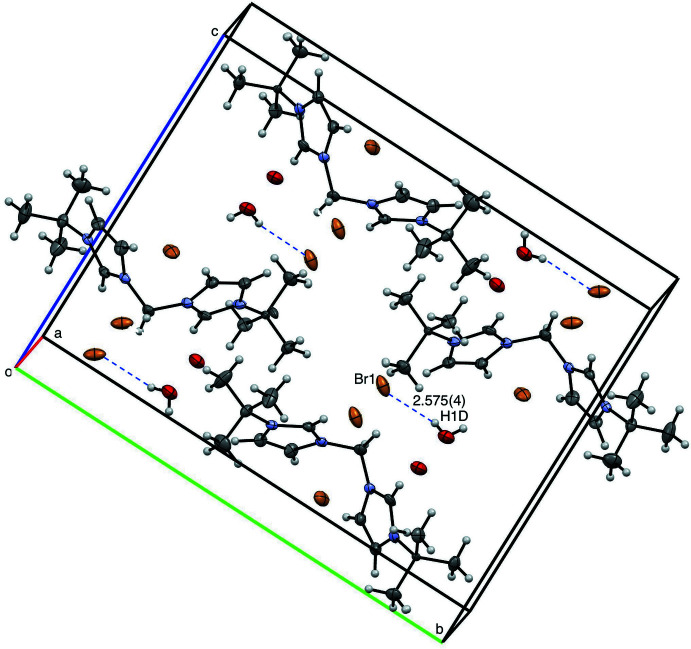
View of four mol­ecules of [^*t*Bu^NHC_2_Me][Br]_2_·H_2_O with 50% probability ellipsoids, highlighting inter­molecular distances. Disordered *tert*-butyl groups are omitted for clarity.

The supra­molecular structure of [^*t*Bu^NHC_2_Me][Br]_2_·H_2_O is stabilized by hydrogen bonding (Fig. 5 ▸, Table 1 ▸). Distances between centroids of neighboring imidazoles are greater than 5 Å, suggesting no π-stacking inter­actions (Janiak, 2000 ▸). Hydrogen bonding between one bromide atom and one water mol­ecule is found with Br1⋯H1*D* having a distance of 2.575 (4) Å. One *tert*-butyl group has positional disorder.

**Figure 6:**
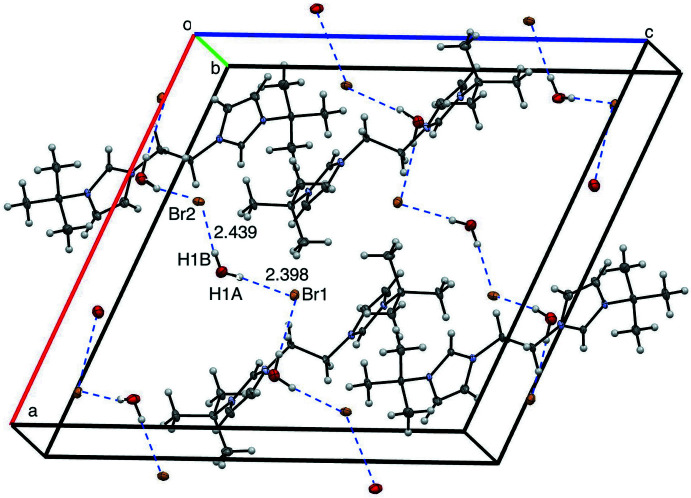
View of four mol­ecules of [^*t*Bu^NHC_2_Et][Br]_2_·2H_2_O with 50% probability ellipsoids, highlighting inter­molecular distances.

The supra­molecular structure of [^*t*Bu^NHC_2_Et][Br]_2_·2H_2_O is stabilized by extensive hydrogen bonding (Fig. 6 ▸, Table 1 ▸). Distances between centroids of neighboring imidazoles are greater than 5 Å, suggesting no π-stacking inter­actions (Janiak, 2000 ▸). Several hydrogen-bonding inter­actions are found between bromide ions and water mol­ecules, including Br2⋯H1*B* (2.439 Å) and Br1⋯H1*A* (2.398 Å).

**Figure 7:**
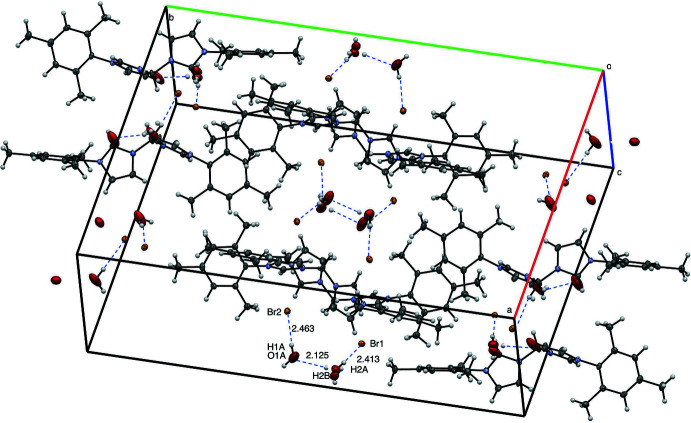
View of eight mol­ecules of [^Mes^NHC_2_Me][Br]_2_·2H_2_O with 50% probability ellipsoids, highlighting inter­molecular distances.

The supra­molecular structure of [^Mes^NHC_2_Me][Br]_2_·2H_2_O is also stabilized by hydrogen bonding (Fig. 7 ▸, Table 1 ▸). No π-stacking inter­actions were found as distances between centroids of aromatic rings of neighboring mol­ecules are greater than 5 Å (Janiak, 2000 ▸). Several hydrogen-bonding inter­actions are observed between bromide ions and water mol­ecules as well as neighboring water mol­ecules, including Br1⋯H2*A* at 2.413 Å, Br2⋯H1*A* at 2.463 Å, and O1*A*⋯H2*B* at 2.125 Å.

**Figure 8:**
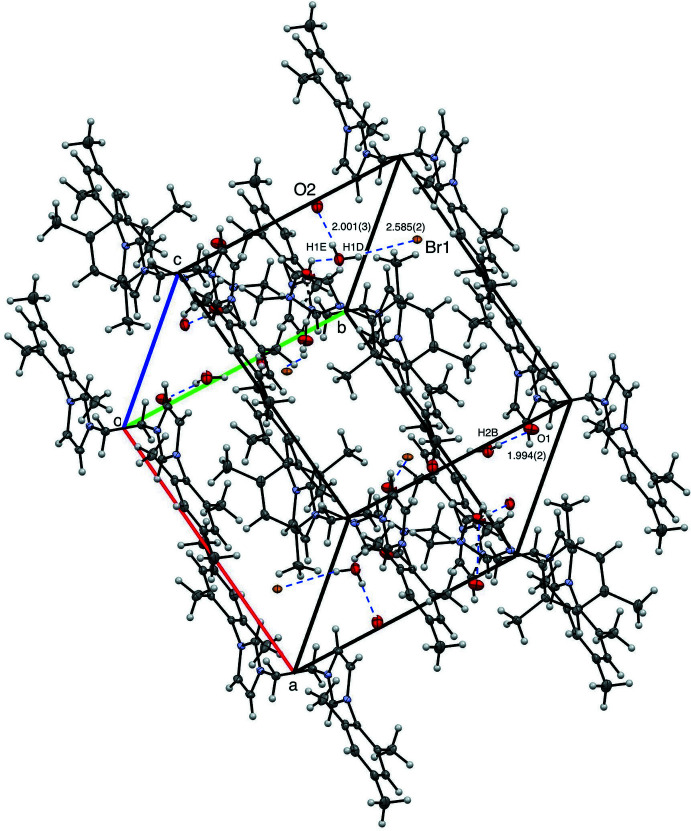
View of eight mol­ecules of [^Mes^NHC_2_Et][Br]_2_·4H_2_O with 50% probability ellipsoids, highlighting inter­molecular distances.

The supra­molecular structure of [^Mes^NHC_2_Et][Br]_2_·4H_2_O is also stabilized by hydrogen bonding (Fig. 8 ▸, Table 1 ▸). No π-stacking is observed between mesityl groups, similar to [^Mes^NHC_2_Me][Br]_2_·2H_2_O as the distance between centroids of the mesityl groups of neighboring fragments is greater than 4.5 Å (Janiak, 2000 ▸). Hydrogen-bonding inter­actions include O1⋯H2*B* at 1.994 (2) Å, O2⋯H1*E* at 2.001 (3) Å, and Br1⋯H1*D* at 2.585 (2) Å.

## 4. Database survey

A survey of the Cambridge Structural Database (Web accessed March 24, 2022; Groom *et al.*, 2016 ▸) and SciFinder (SciFinder, 2022 ▸) yielded no exact matches for the unit cells of [^*t*Bu^NHC_2_Me][Br]_2_·H_2_O, [^*t*Bu^NHC_2_Et][Br]_2_·2H_2_O, or [^Mes^NHC_2_Et][Br]_2_·4H_2_O. A deposited dataset for [^Mes^NHC_2_Me][Br]_2_·2H_2_O was found (Rheingold, 2019 ▸) with a slightly higher *R*1 of 3.94% and data collection at a higher temperature of 150 K, as compared to *R*1 of 3.18% and temperature of 112 K in the current report. As discussed in the introduction, the syntheses of all of the reported structures are reported based on the SciFinder search; however, no additional structural data were found.

## 5. Synthesis and crystallization

**General considerations.** All reagents were purchased from commercial suppliers and used without further purification. ^1^H NMR data were collected on a Varian 400 MHz spectrometer and referenced to residual CHCl_3_.

**Synthesis of 1-** ***tert*** **-butyl-1** ***H*** **-imidazole, (** ***^t^*** **^Bu^Im).** The procedure was adapted from a literature procedure (Liu *et al.*, 2003 ▸). A round-bottom flask was charged with 10.0 mL (95 mmol, 1 eq.) of *tert*-butyl­amine, 11.0 mL of 40% glyoxal (95 mmol, 1 eq.), approximately 100 mL of methanol, and approximately 25 mL of deionized water and a stir bar, then heated to 343 K under reflux. 7.81 mL of 37% formaldehyde (95 mmol, 1 eq.) were added, followed by 3.70 mL of ammonium hydroxide (95 mmol, 1 eq.) added dropwise over 5 minutes while stirring. The solution was refluxed at 343 K for 5 h, resulting in a light red–orange solution. Excess solvent was removed *in vacuo*, and the resulting product was diluted with approximately 150 mL of di­chloro­methane and washed twice with 50 mL of deionized H_2_O until the aqueous layers ran clear. The product was vacuum distilled at ∼373 K, yielding a clear liquid, which was weighed in a tared vial, resulting in 7.95 g (34% yield) of ^*t*Bu^Im, and characterized by ^1^H NMR spectroscopy in CDCl_3_.

**Synthesis of 1-(2,4,6-tri­methyl­phen­yl)-1** ***H*** **-imidazole, (^Mes^Im).** The procedure was adapted from a literature procedure (Liu *et al.*, 2003; Gardiner *et al.*, 1999 ▸). A 250 mL three-neck round-bottom flask was charged with 15.000 g (110.9 mmol, 1 eq.) of 2,4,6-tri­methyl­aniline, 16.090 g (110.9 mmol, 1 eq) of 40% glyoxal, and ∼75 mL of methanol and stirred for 24 h after which the solution turned orange with a yellow precipitate. 11.86 g (221.8 mmol, 2 eq.) of ammonium chloride, 18.00 g (221.8 mmol, 2 eq.) of 37% formaldehyde, and 300 mL of methanol were added, and the solution was refluxed for 24 h at 373 K, at which point the solution was deep brown. After being cooled to room temperature, 25.57 g (221.8 mmol, 2 eq.) of 85% phospho­ric acid were added dropwise over ten minutes and the solution was refluxed for 16 h at 368 K. Excess solvent was removed *in vacuo* at 313 K, and the viscous brown residue was poured over ∼300 g of ice and neutralized to pH 10 with a saturated solution of potassium hydroxide, resulting in a clear solution with a chunky brown precipitate. The product was taken into diethyl ether by washing the solution three times with ∼100 mL of diethyl ether. The diethyl ether solution was washed thrice with ∼100 mL of water, thrice with ∼100 mL of brine, and dried overnight over sodium sulfate. Sodium sulfate solids were gravity filtered from the solution and the solvent was removed *in vacuo* resulting in a brown solid. The product was recrystallized from hot ethyl acetate, resulting in 9.49 g (46% yield) of tan crystals, which were characterized by ^1^H NMR spectroscopy and identified as ^Mes^Im.

**Synthesis of 1,1′-di(** ***tert*** **-but­yl)-3,3′-methyl­ene-diimidazolium dibromide, [** ***^t^*** **^Bu^NHC_2_Me][Br]_2._** 1.850 g (14.9 mmol, 2.5 eq.) of ^*t*bu^Im and 0.4194 mL (5.9 mmol, 1 eq.) of di­bromo­methane, a stir bar, and ∼20 mL of toluene were stirred in a 50 mL round-bottomed flask. The solution was then heated to 423 K and refluxed for 46 h, resulting in the formation of a dark orange–brown solution. The solution was cooled in an ice bath, resulting in a fine white precipitate which was collected *via* vacuum filtration, washed twice with ∼5 mL of cold toluene, filtered and dried. 1.120 g (78.02% yield) of a fine white solid identified as [^*t*Bu^NHC_2_Me][Br]_2_ were isolated. Crystals suitable for X-ray diffraction were obtained by recrystallization from hot methanol. The product was characterized by ^1^H NMR spectroscopy. The ^1^H NMR data were consistent with those previously reported (Scherg *et al.*, 2006 ▸).

**Synthesis of 1,1′-di(** ***tert-*** **but­yl)-3,3′-ethyl­ene-diimidazolium dibromide [** ***^t^*** **^Bu^NHC_2_Et][Br]_2_.** A 250 mL round-bottomed flask was charged with 2.017 g (16.2 mmol, 2.5 eq.) of ^*t*bu^Im, 0.562 mL (6.45 mmol, 1 eq.) of di­bromo­ethane, a stir bar, and ∼20 mL of toluene. The mixture was refluxed at 423 K and stirred for 46 h, at which point the solution was a rusty brown color. The flask was then placed in an ice bath, and the resulting precipitate was collected *via* vacuum filtration and washed twice with ∼5 mL of cold toluene. The resulting solids were dried and weighed, yielding 1.727 g (61% yield) of [^*t*Bu^NHC_2_Et][Br]_2_ and single crystals suitable for X-ray diffraction were obtained *via* recrystallization from hot methanol. ^1^H NMR data were consistent with those previously reported (Scherg *et al.*, 2006 ▸).

**Synthesis of 1,1′-di(mesit­yl)-3,3′-methyl­ene-diimidazolium dibromide, [^Mes^NHC_2_Me][Br]_2_.** The procedure was adapted from a literature procedure (Gardiner *et al.*, 1999 ▸). 5.00 g (26.8 mmol, 2.5 eq.) of ^Mes^Im we added to a 50 mL round-bottomed flask with a stir bar and ∼20 mL of toluene. 0.754 mL (10.72 mmol, 1 eq.) of di­bromo­methane were added and the solution was refluxed at 423 K for 20 h. The solution was cooled in an ice bath, resulting in a white precipitate. The white solid was recrystallized from ∼12 mL of hot methanol. The product was obtained in 17% yield (1.10 g) as tan crystals identified as [^Mes^NHC_2_Me][Br]_2_ suitable for X-ray diffraction and characterized by ^1^H NMR.

**Synthesis of 1,1′-di(mesit­yl)-3,3′-ethyl­ene-diimidazolium dibromide, [^Mes^NHC_2_Et][Br]_2_.** A 250 mL three-neck round-bottom flask was charged with 4.438 g (23.8 mmol, 2.5 eq.) of ^Mes^Im, 0.824 mL (9.52 mmol, 1 eq.) of 1,2-di­bromo­ethane, and ∼20 mL of toluene. The reaction mixture was heated to 423 K and refluxed for 19 h, resulting in a cloudy yellow solution. The solution was cooled in an ice bath and the precipitate was collected and recrystallized from ∼25 mL of hot methanol, resulting in 2.962 g (55% yield) of tan crystals which were analyzed *via* ^1^H NMR spectroscopy and identified as [^Mes^NHC_2_Et][Br]_2_.

## 6. Refinement

Crystal data, data collection and structure refinement details are summarized in Table 2 ▸. Most hydrogen atoms were placed in calculated positions using the AFIX commands of *SHELXL* and included as riding contributions with distances of 0.95 Å for C—H, 0.99 Å for CH_2_ and 0.98 Å for CH_3_. Methyl H atoms were allowed to rotate but not to tip to best fit the experimental electron density. *U*_iso_ values of riding H atoms were set to 1.2 times *U*_eq_(C) for CH and CH_2_, and 1.5 times *U*_eq_(C) for CH_3_ and H_2_O. For [^*t*Bu^NHC_2_Me][Br]_2_, the SADI command of *SHELX* was used to model disorder in one of the *tert*-butyl moieties for N4—C0*AA* and N4—C12, C0*AA*—C00*N* and C14—C12, and C1*AA*—C0*AA* and C13—C12 to restrain distances within a sigma of 0.02 Å. The population parameters for the disordered *tert*-butyl groups are 0.54019 for C12–C14, and 0.45981 for C00*N*, C0*AA*, and C1*AA*. The highest peak and deepest hole are both near a heavy atom Br1 with a distance of 0.88 Å from the highest peak of 1.49 e Å^−3^ and a distance of 0.73 Å from the deepest hole of −1.10 e Å^−3^.

## Supplementary Material

Crystal structure: contains datablock(s) sces01006_0m, est01043_0m, at01019_0ma, est01041d_0ma, global. DOI: 10.1107/S2056989022008003/mw2188sup1.cif

Structure factors: contains datablock(s) sces01006_0m. DOI: 10.1107/S2056989022008003/mw2188sces01006_0msup2.hkl

Structure factors: contains datablock(s) est01043_0m. DOI: 10.1107/S2056989022008003/mw2188est01043_0msup3.hkl

Structure factors: contains datablock(s) at01019_0ma. DOI: 10.1107/S2056989022008003/mw2188at01019_0masup4.hkl

Structure factors: contains datablock(s) est01041d_0ma. DOI: 10.1107/S2056989022008003/mw2188est01041d_0masup5.hkl

Supporting information file. DOI: 10.1107/S2056989022008003/mw2188sces01006_0msup6.cml

Supporting information file. DOI: 10.1107/S2056989022008003/mw2188est01043_0msup7.cml

Supporting information file. DOI: 10.1107/S2056989022008003/mw2188at01019_0masup8.cml

Supporting information file. DOI: 10.1107/S2056989022008003/mw2188est01041d_0masup9.cml

CCDC references: 2195736, 2195735, 2195734, 2195733

Additional supporting information:  crystallographic information; 3D view; checkCIF report

## Figures and Tables

**Table 1:** Inter­molecular distances (Å) in the unit cells of [^*R*^NHC_2_ *R*_1_][*X*]_2_·*n*H_2_O Standard deviations for distances including some H atoms are omitted where H atoms were positionally fixed.

Compound	Atoms	Distance
[*^*t*^* ^Bu^NHC_2_Me][Br]_2_·H_2_O	Br1⋯H1*D*	2.575 (4)
[*^*t*^* ^Bu^NHC_2_Et][Br]_2_·2H_2_O	Br1⋯H1*A*	2.398
	Br2⋯H1*B*	2.439
[^Mes^NHC_2_Me][Br]_2_·2H_2_O	Br1⋯H2*A*	2.413
	Br2⋯H1*A*	2.463
	O1*A*⋯H2*B*	2.125
[^Mes^NHC_2_Et][Br]_2_·4H_2_O	O1⋯H2*B*	1.994 (2)
	O2⋯H1*E*	2.001 (3)
	Br1⋯H1*D*	2.585 (2)

**Table 2:** Experimental details

	[^Mes^NHC_2_Me][Br]_2_·2H_2_O	[^*t*Bu^NHC_2_Me][Br]_2_·H_2_O	[^*t*Bu^NHC_2_Et][Br]_2_·2H_2_O	[^Mes^NHC_2_Et][Br]_2_·4H_2_O
Crystal data				
Chemical formula	C_25_H_30_N_4_^2+^·2Br^−^·2H_2_O	C_15_H_26_N_4_^+^·2Br^−^·H_2_O	C_16_H_28_N_4_^2+^·2Br^−^·2H_2_O	C_26_H_32_N_4_^2+^·2Br^−^·4H_2_O
*M*_r_	582.38	440.23	472.27	632.42
Crystal system, space group	Orthorhombic, *P* *c* *c* *n*	Monoclinic, *P*2_1_/*c*	Monoclinic, *P*2_1_/*n*	Monoclinic, *P*2_1_/*c*
Temperature (K)	112	293	100	100
*a*, *b*, *c* (Å)	21.5695 (6), 28.3385 (6), 8.9401 (2)	7.211 (5), 18.311 (17), 15.409 (5)	17.1577 (6), 7.3180 (2), 18.2712 (6)	12.4230 (3), 13.1447 (3), 9.2780 (2)
α, β, γ (°)	90, 90, 90	90, 101.35 (3), 90	90, 112.786 (1), 90	90, 108.379 (1), 90
*V* (Å^3^)	5464.6 (2)	1995 (2)	2115.09 (12)	1437.78 (6)
*Z*	8	4	4	2
Radiation type	Mo *K*α	Mo *K*α	Mo *K*α	Mo *K*α
μ (mm^−1^)	2.99	4.07	3.85	2.86
Crystal size (mm)	0.4 × 0.3 × 0.25	0.3 × 0.15 × 0.1	0.2 × 0.1 × 0.05	0.15 × 0.15 × 0.05

Data collection				
Diffractometer	Bruker Venture D8 Kappa	Bruker APEXII CCD	Bruker Venture D8 Kappa	Bruker Venture D8 Kappa
Absorption correction	Multi-scan (*SADABS*; Bruker, 2016 ▸)	Multi-scan (*SADABS*; Bruker, 2016 ▸)	Multi-scan (*SADABS*; Bruker, 2016 ▸)	Multi-scan (*SADABS*; Bruker, 2016 ▸)
*T*_min_, *T*_max_	0.386, 0.748	0.544, 0.747	0.496, 0.748	0.544, 0.750
No. of measured, independent and observed [*I* > 2σ(*I*)] reflections	39085, 5954, 5530	33565, 4402, 3780	31474, 4664, 4168	28199, 3165, 3028
*R*_int_	0.035	0.043	0.059	0.025
(sin θ/λ)_max_ (Å^−1^)	0.641	0.641	0.641	0.641

Refinement				
*R*[*F* ^2^ > 2σ(*F* ^2^)], *wR*(*F* ^2^), *S*	0.032, 0.066, 1.19	0.035, 0.068, 1.08	0.031, 0.066, 1.11	0.018, 0.044, 1.10
No. of reflections	5954	4402	4664	3165
No. of parameters	338	259	253	194
No. of restraints	0	3	0	0
H-atom treatment	H atoms treated by a mixture of independent and constrained refinement	H atoms treated by a mixture of independent and constrained refinement	H atoms treated by a mixture of independent and constrained refinement	H atoms treated by a mixture of independent and constrained refinement
Δρ_max_, Δρ_min_ (e Å^−3^)	0.42, −0.54	1.49, −1.10	0.60, −0.61	0.33, −0.29

Computer programs: *APEX3* and *SAINT* (Bruker, 2016 ▸), *olex2.solve* (Bourhis *et al.*, 2015 ▸), *SHELXT* (Sheldrick, 2015*a* ▸), *SHELXL* (Sheldrick, 2015*b* ▸), and *OLEX2* (Dolomanov *et al.*, 2009 ▸).

## supplementary crystallographic information

### 1,1'-Methylenebis(3-tert-butylimidazolium) dibromide monohydrate (sces01006_0m) Crystal data

**Table:**

C_15_H_26_N_4_^+^·2Br^−^·H_2_O	*F*(000) = 896
*M**_r_* = 440.23	*D*_x_ = 1.466 Mg m^−^^3^
Monoclinic, *P*2_1_/*c*	Mo *K*α radiation, λ = 0.71073 Å
*a* = 7.211 (5) Å	Cell parameters from 9889 reflections
*b* = 18.311 (17) Å	θ = 2.6–30.2°
*c* = 15.409 (5) Å	µ = 4.07 mm^−^^1^
β = 101.35 (3)°	*T* = 293 K
*V* = 1995 (2) Å^3^	Prism, clear colourless
*Z* = 4	0.3 × 0.15 × 0.1 mm

### 1,1'-Methylenebis(3-tert-butylimidazolium) dibromide monohydrate (sces01006_0m) Data collection

**Table:**

Bruker APEXII CCD diffractometer	3780 reflections with *I* > 2σ(*I*)
φ and ω scans	*R*_int_ = 0.043
Absorption correction: multi-scan (SADABS; Bruker, 2016)	θ_max_ = 27.1°, θ_min_ = 2.7°
*T*_min_ = 0.544, *T*_max_ = 0.747	*h* = −9→8
33565 measured reflections	*k* = −23→23
4402 independent reflections	*l* = −19→19

### 1,1'-Methylenebis(3-tert-butylimidazolium) dibromide monohydrate (sces01006_0m) Refinement

**Table:**

Refinement on *F*^2^	Primary atom site location: dual
Least-squares matrix: full	Hydrogen site location: mixed
*R*[*F*^2^ > 2σ(*F*^2^)] = 0.035	H atoms treated by a mixture of independent and constrained refinement
*wR*(*F*^2^) = 0.068	*w* = 1/[σ^2^(*F*_o_^2^) + (0.0006*P*)^2^ + 4.1194*P*] where *P* = (*F*_o_^2^ + 2*F*_c_^2^)/3
*S* = 1.08	(Δ/σ)_max_ = 0.001
4402 reflections	Δρ_max_ = 1.49 e Å^−^^3^
259 parameters	Δρ_min_ = −1.10 e Å^−^^3^
3 restraints	

### 1,1'-Methylenebis(3-tert-butylimidazolium) dibromide monohydrate (sces01006_0m) Special details

**Table:**

Geometry. All esds (except the esd in the dihedral angle between two l.s. planes) are estimated using the full covariance matrix. The cell esds are taken into account individually in the estimation of esds in distances, angles and torsion angles; correlations between esds in cell parameters are only used when they are defined by crystal symmetry. An approximate (isotropic) treatment of cell esds is used for estimating esds involving l.s. planes.

### 1,1'-Methylenebis(3-tert-butylimidazolium) dibromide monohydrate (sces01006_0m) Fractional atomic coordinates and isotropic or equivalent isotropic displacement parameters (Å^2^)

**Table:**

	*x*	*y*	*z*	*U*_iso_*/*U*_eq_	Occ. (<1)
Br2	0.83773 (4)	0.86331 (2)	0.55378 (2)	0.03323 (9)	
Br1	1.05299 (4)	0.61882 (2)	0.35706 (2)	0.03727 (10)	
N3	0.4514 (3)	0.71177 (11)	0.18236 (13)	0.0175 (4)	
N1	0.5823 (3)	0.48054 (11)	0.16964 (13)	0.0162 (4)	
O1	0.8362 (4)	0.78047 (16)	0.35775 (17)	0.0419 (6)	
N2	0.4742 (3)	0.58205 (11)	0.21324 (13)	0.0170 (4)	
N4	0.5288 (3)	0.80162 (12)	0.10500 (14)	0.0225 (5)	
C1	0.6311 (4)	0.54357 (13)	0.21172 (16)	0.0181 (5)	
H5	0.753852	0.558228	0.235943	0.022*	
C5	0.2905 (4)	0.73014 (14)	0.12095 (18)	0.0221 (5)	
C4	0.5935 (4)	0.75572 (14)	0.17061 (16)	0.0211 (5)	
H9	0.716674	0.754245	0.203041	0.025*	
C7	0.4681 (4)	0.65531 (14)	0.25048 (16)	0.0212 (5)	
H8A	0.361201	0.658686	0.279890	0.025*	
H8B	0.582372	0.663573	0.294434	0.025*	
C8	0.7152 (4)	0.42139 (14)	0.15085 (18)	0.0207 (5)	
C6	0.3396 (4)	0.78602 (15)	0.07277 (19)	0.0251 (6)	
C2	0.3869 (4)	0.47957 (15)	0.14242 (18)	0.0216 (5)	
C3	0.3195 (4)	0.54263 (15)	0.16910 (19)	0.0239 (6)	
C13	0.5981 (4)	0.86039 (16)	−0.02694 (18)	0.0290 (6)	
H15A	0.613085	0.812884	−0.050991	0.043*	0.54 (3)
H15B	0.674562	0.895007	−0.050884	0.043*	0.54 (3)
H15C	0.467761	0.874789	−0.042171	0.043*	0.54 (3)
H15D	0.641328	0.812747	−0.039773	0.043*	0.46 (3)
H15E	0.674290	0.896891	−0.047847	0.043*	0.46 (3)
H15F	0.468475	0.866370	−0.055952	0.043*	0.46 (3)
C12	0.6629 (17)	0.8579 (5)	0.0781 (8)	0.014 (2)	0.54 (3)
C11	0.6634 (5)	0.35011 (16)	0.1920 (2)	0.0402 (8)	
H3A	0.676730	0.356294	0.254815	0.060*	
H3B	0.746068	0.311841	0.180278	0.060*	
H3C	0.534897	0.337462	0.166825	0.060*	
C10	0.9155 (4)	0.44460 (17)	0.1903 (3)	0.0408 (8)	
H1A	0.944282	0.489150	0.162880	0.061*	
H1B	1.002017	0.407121	0.180300	0.061*	
H1C	0.927348	0.452256	0.252799	0.061*	
C9	0.6908 (5)	0.4157 (2)	0.0510 (2)	0.0399 (8)	
H4A	0.564299	0.400122	0.026305	0.060*	
H4B	0.779299	0.380851	0.036360	0.060*	
H4C	0.713383	0.462547	0.027048	0.060*	
C15	0.8712 (15)	0.8362 (6)	0.1050 (8)	0.0245 (19)	0.54 (3)
H13A	0.906281	0.834192	0.168381	0.037*	0.54 (3)
H13B	0.948184	0.871611	0.082775	0.037*	0.54 (3)
H13C	0.889802	0.789067	0.080737	0.037*	0.54 (3)
C14	0.6197 (18)	0.9291 (5)	0.1206 (7)	0.025 (2)	0.54 (3)
H14A	0.487306	0.939908	0.103235	0.038*	0.54 (3)
H14B	0.692055	0.967891	0.101603	0.038*	0.54 (3)
H14C	0.652454	0.924641	0.183859	0.038*	0.54 (3)
H1D	0.905 (6)	0.746 (2)	0.365 (3)	0.061 (15)*	
C14A	0.530 (3)	0.9398 (5)	0.0928 (10)	0.034 (3)	0.46 (3)
H00A	0.398530	0.942603	0.064831	0.050*	0.46 (3)
H00B	0.596718	0.980230	0.074007	0.050*	0.46 (3)
H00C	0.540922	0.941501	0.155917	0.050*	0.46 (3)
C12A	0.614 (2)	0.8681 (6)	0.0668 (11)	0.021 (3)	0.46 (3)
C15A	0.823 (2)	0.8614 (11)	0.1096 (10)	0.038 (3)	0.46 (3)
H1AA	0.837624	0.866529	0.172622	0.057*	0.46 (3)
H1AB	0.893103	0.898947	0.087072	0.057*	0.46 (3)
H1AC	0.868755	0.814380	0.096076	0.057*	0.46 (3)
H1E	0.839 (5)	0.800 (2)	0.399 (3)	0.041 (12)*	
H6	0.331 (4)	0.4388 (16)	0.1108 (19)	0.023 (7)*	
H11	0.270 (5)	0.8115 (18)	0.027 (2)	0.035 (9)*	
H10	0.175 (5)	0.7062 (18)	0.118 (2)	0.037 (9)*	
H7	0.201 (5)	0.5612 (17)	0.164 (2)	0.034 (9)*	

### 1,1'-Methylenebis(3-tert-butylimidazolium) dibromide monohydrate (sces01006_0m) Atomic displacement parameters (Å^2^)

**Table:**

	*U*^11^	*U*^22^	*U*^33^	*U*^12^	*U*^13^	*U*^23^
Br2	0.02663 (15)	0.03928 (17)	0.02985 (16)	0.01469 (13)	−0.00401 (11)	−0.00144 (13)
Br1	0.02382 (15)	0.0520 (2)	0.03702 (18)	−0.01154 (13)	0.00837 (12)	−0.02086 (14)
N3	0.0203 (11)	0.0185 (10)	0.0146 (10)	−0.0049 (8)	0.0058 (8)	−0.0011 (8)
N1	0.0134 (10)	0.0203 (11)	0.0140 (10)	−0.0024 (8)	0.0008 (8)	0.0046 (8)
O1	0.0559 (17)	0.0417 (15)	0.0233 (13)	−0.0041 (13)	−0.0040 (11)	0.0034 (11)
N2	0.0182 (11)	0.0207 (11)	0.0125 (10)	−0.0039 (8)	0.0040 (8)	0.0026 (8)
N4	0.0315 (13)	0.0209 (11)	0.0153 (11)	−0.0117 (9)	0.0053 (9)	−0.0034 (8)
C1	0.0149 (12)	0.0223 (13)	0.0156 (12)	−0.0039 (10)	−0.0009 (9)	0.0041 (10)
C5	0.0210 (14)	0.0194 (13)	0.0241 (14)	−0.0026 (10)	0.0002 (11)	−0.0025 (10)
C4	0.0243 (14)	0.0283 (14)	0.0106 (12)	−0.0092 (11)	0.0035 (10)	−0.0022 (10)
C7	0.0285 (14)	0.0233 (13)	0.0135 (12)	−0.0036 (11)	0.0083 (10)	0.0014 (10)
C8	0.0176 (13)	0.0180 (12)	0.0271 (14)	−0.0015 (10)	0.0060 (11)	0.0024 (10)
C6	0.0304 (15)	0.0196 (13)	0.0227 (14)	−0.0033 (11)	−0.0010 (12)	−0.0013 (11)
C2	0.0126 (12)	0.0246 (14)	0.0266 (14)	−0.0068 (10)	0.0016 (10)	0.0013 (11)
C3	0.0136 (13)	0.0256 (14)	0.0328 (16)	−0.0051 (11)	0.0053 (11)	0.0022 (11)
C13	0.0353 (16)	0.0344 (16)	0.0195 (14)	−0.0012 (13)	0.0109 (12)	0.0030 (12)
C12	0.023 (5)	0.011 (3)	0.008 (4)	−0.003 (3)	0.002 (4)	0.000 (3)
C11	0.0429 (19)	0.0235 (15)	0.060 (2)	0.0033 (13)	0.0254 (17)	0.0115 (14)
C10	0.0166 (15)	0.0285 (16)	0.072 (2)	0.0027 (12)	−0.0039 (15)	−0.0055 (16)
C9	0.0406 (19)	0.050 (2)	0.0316 (17)	0.0052 (15)	0.0133 (14)	−0.0072 (15)
C15	0.014 (4)	0.028 (5)	0.031 (3)	−0.009 (3)	0.003 (3)	0.000 (3)
C14	0.032 (5)	0.020 (3)	0.028 (4)	−0.006 (3)	0.014 (3)	−0.002 (3)
C14A	0.066 (9)	0.011 (3)	0.030 (5)	−0.003 (4)	0.026 (6)	0.000 (3)
C12A	0.025 (6)	0.022 (5)	0.014 (4)	−0.005 (4)	−0.004 (4)	0.008 (3)
C15A	0.028 (6)	0.047 (8)	0.032 (5)	−0.018 (5)	−0.010 (5)	0.022 (5)

### 1,1'-Methylenebis(3-tert-butylimidazolium) dibromide monohydrate (sces01006_0m) Geometric parameters (Å, º)

**Table:**

N3—C5	1.387 (3)	C13—H15D	0.9600
N3—C4	1.343 (3)	C13—H15E	0.9600
N3—C7	1.461 (3)	C13—H15F	0.9600
N1—C1	1.337 (3)	C13—C12	1.595 (13)
N1—C8	1.512 (3)	C13—C12A	1.434 (16)
N1—C2	1.389 (3)	C12—C15	1.530 (10)
O1—H1D	0.80 (4)	C12—C14	1.519 (10)
O1—H1E	0.73 (4)	C11—H3A	0.9600
N2—C1	1.337 (3)	C11—H3B	0.9600
N2—C7	1.463 (3)	C11—H3C	0.9600
N2—C3	1.389 (3)	C10—H1A	0.9600
N4—C4	1.327 (3)	C10—H1B	0.9600
N4—C6	1.387 (4)	C10—H1C	0.9600
N4—C12	1.525 (9)	C9—H4A	0.9600
N4—C12A	1.531 (11)	C9—H4B	0.9600
C1—H5	0.9300	C9—H4C	0.9600
C5—C6	1.352 (4)	C15—H13A	0.9600
C5—H10	0.94 (3)	C15—H13B	0.9600
C4—H9	0.9300	C15—H13C	0.9600
C7—H8A	0.9700	C14—H14A	0.9600
C7—H8B	0.9700	C14—H14B	0.9600
C8—C11	1.529 (4)	C14—H14C	0.9600
C8—C10	1.514 (4)	C14A—H00A	0.9600
C8—C9	1.518 (4)	C14A—H00B	0.9600
C6—H11	0.91 (3)	C14A—H00C	0.9600
C2—C3	1.348 (4)	C14A—C12A	1.531 (12)
C2—H6	0.94 (3)	C12A—C15A	1.527 (11)
C3—H7	0.91 (3)	C15A—H1AA	0.9600
C13—H15A	0.9600	C15A—H1AB	0.9600
C13—H15B	0.9600	C15A—H1AC	0.9600
C13—H15C	0.9600		
			
C5—N3—C7	127.0 (2)	C12A—C13—H15F	109.5
C4—N3—C5	108.7 (2)	N4—C12—C13	102.7 (7)
C4—N3—C7	124.3 (2)	N4—C12—C15	113.1 (8)
C1—N1—C8	126.5 (2)	C15—C12—C13	111.0 (8)
C1—N1—C2	108.2 (2)	C14—C12—N4	105.6 (7)
C2—N1—C8	125.2 (2)	C14—C12—C13	111.6 (6)
H1D—O1—H1E	112 (4)	C14—C12—C15	112.4 (7)
C1—N2—C7	125.5 (2)	C8—C11—H3A	109.5
C1—N2—C3	108.6 (2)	C8—C11—H3B	109.5
C3—N2—C7	125.8 (2)	C8—C11—H3C	109.5
C4—N4—C6	108.4 (2)	H3A—C11—H3B	109.5
C4—N4—C12	119.2 (6)	H3A—C11—H3C	109.5
C4—N4—C12A	133.6 (7)	H3B—C11—H3C	109.5
C6—N4—C12	132.4 (6)	C8—C10—H1A	109.5
C6—N4—C12A	117.7 (7)	C8—C10—H1B	109.5
N1—C1—N2	108.7 (2)	C8—C10—H1C	109.5
N1—C1—H5	125.6	H1A—C10—H1B	109.5
N2—C1—H5	125.6	H1A—C10—H1C	109.5
N3—C5—H10	123 (2)	H1B—C10—H1C	109.5
C6—C5—N3	106.5 (2)	C8—C9—H4A	109.5
C6—C5—H10	131 (2)	C8—C9—H4B	109.5
N3—C4—H9	125.7	C8—C9—H4C	109.5
N4—C4—N3	108.6 (2)	H4A—C9—H4B	109.5
N4—C4—H9	125.7	H4A—C9—H4C	109.5
N3—C7—N2	111.8 (2)	H4B—C9—H4C	109.5
N3—C7—H8A	109.3	C12—C15—H13A	109.5
N3—C7—H8B	109.3	C12—C15—H13B	109.5
N2—C7—H8A	109.3	C12—C15—H13C	109.5
N2—C7—H8B	109.3	H13A—C15—H13B	109.5
H8A—C7—H8B	107.9	H13A—C15—H13C	109.5
N1—C8—C11	108.4 (2)	H13B—C15—H13C	109.5
N1—C8—C10	108.2 (2)	C12—C14—H14A	109.5
N1—C8—C9	107.0 (2)	C12—C14—H14B	109.5
C10—C8—C11	111.4 (3)	C12—C14—H14C	109.5
C10—C8—C9	109.7 (3)	H14A—C14—H14B	109.5
C9—C8—C11	111.9 (3)	H14A—C14—H14C	109.5
N4—C6—H11	122 (2)	H14B—C14—H14C	109.5
C5—C6—N4	107.7 (2)	H00A—C14A—H00B	109.5
C5—C6—H11	131 (2)	H00A—C14A—H00C	109.5
N1—C2—H6	118.1 (18)	H00B—C14A—H00C	109.5
C3—C2—N1	107.6 (2)	C12A—C14A—H00A	109.5
C3—C2—H6	134.3 (18)	C12A—C14A—H00B	109.5
N2—C3—H7	120 (2)	C12A—C14A—H00C	109.5
C2—C3—N2	106.9 (2)	N4—C12A—C14A	111.9 (9)
C2—C3—H7	133 (2)	C13—C12A—N4	110.5 (9)
H15A—C13—H15B	109.5	C13—C12A—C14A	113.1 (8)
H15A—C13—H15C	109.5	C13—C12A—C15A	107.5 (10)
H15B—C13—H15C	109.5	C15A—C12A—N4	101.8 (9)
H15D—C13—H15E	109.5	C15A—C12A—C14A	111.4 (10)
H15D—C13—H15F	109.5	C12A—C15A—H1AA	109.5
H15E—C13—H15F	109.5	C12A—C15A—H1AB	109.5
C12—C13—H15A	109.5	C12A—C15A—H1AC	109.5
C12—C13—H15B	109.5	H1AA—C15A—H1AB	109.5
C12—C13—H15C	109.5	H1AA—C15A—H1AC	109.5
C12A—C13—H15D	109.5	H1AB—C15A—H1AC	109.5
C12A—C13—H15E	109.5		
			
N3—C5—C6—N4	−0.3 (3)	C7—N2—C1—N1	−177.6 (2)
N1—C2—C3—N2	−0.4 (3)	C7—N2—C3—C2	177.5 (2)
C1—N1—C8—C11	122.5 (3)	C8—N1—C1—N2	178.4 (2)
C1—N1—C8—C10	1.6 (3)	C8—N1—C2—C3	−177.9 (2)
C1—N1—C8—C9	−116.6 (3)	C6—N4—C4—N3	−0.8 (3)
C1—N1—C2—C3	−0.3 (3)	C6—N4—C12—C13	−36.8 (9)
C1—N2—C7—N3	97.2 (3)	C6—N4—C12—C15	−156.5 (6)
C1—N2—C3—C2	0.9 (3)	C6—N4—C12—C14	80.3 (8)
C5—N3—C4—N4	0.6 (3)	C6—N4—C12A—C13	−59.5 (11)
C5—N3—C7—N2	75.7 (3)	C6—N4—C12A—C14A	67.4 (12)
C4—N3—C5—C6	−0.2 (3)	C6—N4—C12A—C15A	−173.5 (9)
C4—N3—C7—N2	−105.5 (3)	C2—N1—C1—N2	0.8 (3)
C4—N4—C6—C5	0.7 (3)	C2—N1—C8—C11	−60.3 (3)
C4—N4—C12—C13	142.2 (5)	C2—N1—C8—C10	178.8 (3)
C4—N4—C12—C15	22.6 (10)	C2—N1—C8—C9	60.6 (3)
C4—N4—C12—C14	−100.7 (6)	C3—N2—C1—N1	−1.0 (3)
C4—N4—C12A—C13	127.1 (8)	C3—N2—C7—N3	−78.8 (3)
C4—N4—C12A—C14A	−105.9 (8)	C12—N4—C4—N3	179.9 (5)
C4—N4—C12A—C15A	13.1 (14)	C12—N4—C6—C5	179.8 (6)
C7—N3—C5—C6	178.7 (2)	C12A—N4—C4—N3	173.0 (8)
C7—N3—C4—N4	−178.3 (2)	C12A—N4—C6—C5	−174.3 (7)

### 1,1'-(Ethane-1,2-diyl)bis(3-tert-butylimidazolium) dibromide dihydrate (est01043_0m) Crystal data

**Table:**

C_16_H_28_N_4_^2+^·2Br^−^·2H_2_O	*F*(000) = 968
*M**_r_* = 472.27	*D*_x_ = 1.483 Mg m^−^^3^
Monoclinic, *P*2_1_/*n*	Mo *K*α radiation, λ = 0.71073 Å
*a* = 17.1577 (6) Å	Cell parameters from 9074 reflections
*b* = 7.3180 (2) Å	θ = 2.4–38.4°
*c* = 18.2712 (6) Å	µ = 3.85 mm^−^^1^
β = 112.786 (1)°	*T* = 100 K
*V* = 2115.09 (12) Å^3^	Prism, clear colourless
*Z* = 4	0.2 × 0.1 × 0.05 mm

### 1,1'-(Ethane-1,2-diyl)bis(3-tert-butylimidazolium) dibromide dihydrate (est01043_0m) Data collection

**Table:**

Bruker Venture D8 Kappa diffractometer	4168 reflections with *I* > 2σ(*I*)
φ and ω scans	*R*_int_ = 0.059
Absorption correction: multi-scan (SADABS; Bruker, 2016)	θ_max_ = 27.1°, θ_min_ = 2.6°
*T*_min_ = 0.496, *T*_max_ = 0.748	*h* = −21→21
31474 measured reflections	*k* = −9→9
4664 independent reflections	*l* = −23→23

### 1,1'-(Ethane-1,2-diyl)bis(3-tert-butylimidazolium) dibromide dihydrate (est01043_0m) Refinement

**Table:**

Refinement on *F*^2^	Primary atom site location: dual
Least-squares matrix: full	Hydrogen site location: mixed
*R*[*F*^2^ > 2σ(*F*^2^)] = 0.031	H atoms treated by a mixture of independent and constrained refinement
*wR*(*F*^2^) = 0.066	*w* = 1/[σ^2^(*F*_o_^2^) + (0.013*P*)^2^ + 2.745*P*] where *P* = (*F*_o_^2^ + 2*F*_c_^2^)/3
*S* = 1.11	(Δ/σ)_max_ = 0.001
4664 reflections	Δρ_max_ = 0.60 e Å^−^^3^
253 parameters	Δρ_min_ = −0.61 e Å^−^^3^
0 restraints	

### 1,1'-(Ethane-1,2-diyl)bis(3-tert-butylimidazolium) dibromide dihydrate (est01043_0m) Special details

**Table:**

Geometry. All esds (except the esd in the dihedral angle between two l.s. planes) are estimated using the full covariance matrix. The cell esds are taken into account individually in the estimation of esds in distances, angles and torsion angles; correlations between esds in cell parameters are only used when they are defined by crystal symmetry. An approximate (isotropic) treatment of cell esds is used for estimating esds involving l.s. planes.

### 1,1'-(Ethane-1,2-diyl)bis(3-tert-butylimidazolium) dibromide dihydrate (est01043_0m) Fractional atomic coordinates and isotropic or equivalent isotropic displacement parameters (Å^2^)

**Table:**

	*x*	*y*	*z*	*U*_iso_*/*U*_eq_	
Br2	0.42021 (2)	1.06846 (3)	0.17436 (2)	0.01794 (7)	
Br1	0.64204 (2)	1.22356 (3)	0.46410 (2)	0.01716 (7)	
O2	0.60635 (11)	0.9669 (3)	0.30798 (12)	0.0253 (4)	
H2C	0.612198	1.048204	0.344458	0.038*	
H2D	0.556728	0.988924	0.271398	0.038*	
O1	0.84726 (12)	1.2201 (3)	0.50226 (12)	0.0273 (4)	
H1C	0.874519	1.260282	0.550269	0.041*	
H1D	0.793229	1.222132	0.491369	0.041*	
N1	0.63968 (11)	0.5405 (3)	0.63652 (11)	0.0097 (4)	
N2	0.70265 (12)	0.7072 (3)	0.57816 (11)	0.0111 (4)	
N3	0.79306 (12)	0.7383 (3)	0.42286 (12)	0.0113 (4)	
N4	0.86030 (11)	0.8912 (2)	0.36416 (11)	0.0103 (4)	
C1	0.70549 (14)	0.5497 (3)	0.61555 (13)	0.0100 (4)	
C9	0.61678 (14)	0.3834 (3)	0.67751 (14)	0.0119 (5)	
C7	0.76212 (14)	0.7620 (3)	0.54214 (14)	0.0123 (5)	
H1A	0.818644	0.709890	0.572987	0.015*	
H1B	0.767285	0.896775	0.543092	0.015*	
C4	0.79441 (14)	0.8939 (3)	0.38544 (13)	0.0108 (4)	
C3	0.63152 (15)	0.8025 (3)	0.57441 (15)	0.0152 (5)	
C10	0.68416 (16)	0.2364 (3)	0.69468 (15)	0.0165 (5)	
H9A	0.688285	0.198067	0.644913	0.025*	
H9B	0.668875	0.131113	0.719524	0.025*	
H9C	0.738750	0.285198	0.730682	0.025*	
C2	0.59238 (15)	0.6988 (3)	0.61096 (15)	0.0154 (5)	
C8	0.73079 (14)	0.6942 (3)	0.45708 (14)	0.0133 (5)	
H2A	0.721968	0.560336	0.455808	0.016*	
H2B	0.676038	0.752701	0.425349	0.016*	
C11	0.61411 (16)	0.4549 (3)	0.75498 (15)	0.0176 (5)	
H7A	0.670172	0.500229	0.789205	0.026*	
H7B	0.597527	0.355834	0.782054	0.026*	
H7C	0.572921	0.554573	0.743410	0.026*	
C5	0.86096 (15)	0.6322 (3)	0.42626 (15)	0.0155 (5)	
C12	0.53083 (15)	0.3119 (4)	0.62096 (16)	0.0196 (5)	
H8A	0.488167	0.407825	0.611530	0.029*	
H8B	0.515078	0.205199	0.644608	0.029*	
H8C	0.534144	0.276943	0.570466	0.029*	
C16	0.81928 (16)	1.1913 (3)	0.30107 (15)	0.0170 (5)	
H16A	0.764854	1.140422	0.265770	0.025*	
H16B	0.835344	1.291111	0.273954	0.025*	
H16C	0.814374	1.238204	0.349352	0.025*	
C13	0.88640 (14)	1.0428 (3)	0.32301 (14)	0.0126 (5)	
C15	0.89362 (16)	0.9645 (3)	0.24873 (15)	0.0186 (5)	
H15A	0.935772	0.866340	0.263706	0.028*	
H15B	0.911020	1.061099	0.221125	0.028*	
H15C	0.838699	0.915498	0.213542	0.028*	
C6	0.90270 (15)	0.7277 (3)	0.38943 (15)	0.0157 (5)	
C14	0.97108 (15)	1.1159 (4)	0.38186 (16)	0.0195 (5)	
H14A	0.964657	1.157067	0.430264	0.029*	
H14B	0.989184	1.218801	0.357887	0.029*	
H14C	1.013621	1.018734	0.395112	0.029*	
H5	0.7461 (15)	0.464 (3)	0.6249 (15)	0.006 (6)*	
H4	0.5430 (18)	0.717 (4)	0.6210 (17)	0.023 (8)*	
H12	0.7552 (15)	0.985 (3)	0.3754 (14)	0.003 (6)*	
H11	0.9505 (17)	0.698 (4)	0.3796 (16)	0.018 (7)*	
H3	0.6180 (17)	0.910 (4)	0.5485 (17)	0.018 (7)*	
H10	0.8680 (18)	0.521 (4)	0.4505 (18)	0.028 (8)*	

### 1,1'-(Ethane-1,2-diyl)bis(3-tert-butylimidazolium) dibromide dihydrate (est01043_0m) Atomic displacement parameters (Å^2^)

**Table:**

	*U*^11^	*U*^22^	*U*^33^	*U*^12^	*U*^13^	*U*^23^
Br2	0.01224 (11)	0.01866 (13)	0.02260 (14)	−0.00149 (9)	0.00642 (10)	0.00053 (10)
Br1	0.02064 (13)	0.01617 (12)	0.01448 (13)	−0.00222 (9)	0.00658 (10)	0.00156 (9)
O2	0.0199 (9)	0.0299 (11)	0.0227 (11)	0.0067 (8)	0.0046 (8)	−0.0070 (8)
O1	0.0261 (10)	0.0346 (11)	0.0234 (11)	0.0008 (9)	0.0120 (8)	−0.0035 (9)
N1	0.0115 (9)	0.0117 (9)	0.0082 (9)	−0.0001 (7)	0.0063 (7)	0.0017 (7)
N2	0.0120 (9)	0.0141 (9)	0.0088 (10)	−0.0008 (7)	0.0059 (8)	0.0006 (8)
N3	0.0135 (9)	0.0113 (9)	0.0118 (10)	−0.0022 (7)	0.0078 (8)	0.0015 (7)
N4	0.0129 (9)	0.0103 (9)	0.0098 (10)	−0.0010 (7)	0.0068 (8)	0.0018 (7)
C1	0.0114 (10)	0.0116 (11)	0.0084 (11)	0.0009 (9)	0.0054 (9)	0.0002 (8)
C9	0.0148 (11)	0.0132 (11)	0.0103 (11)	−0.0025 (9)	0.0078 (9)	0.0027 (9)
C7	0.0146 (11)	0.0155 (11)	0.0094 (11)	−0.0034 (9)	0.0075 (9)	0.0031 (9)
C4	0.0125 (10)	0.0127 (11)	0.0087 (11)	−0.0010 (9)	0.0056 (9)	0.0005 (9)
C3	0.0170 (12)	0.0142 (12)	0.0157 (13)	0.0041 (9)	0.0079 (10)	0.0038 (10)
C10	0.0214 (12)	0.0145 (11)	0.0168 (13)	0.0004 (9)	0.0110 (10)	0.0029 (10)
C2	0.0150 (11)	0.0157 (12)	0.0182 (13)	0.0048 (9)	0.0094 (10)	0.0032 (10)
C8	0.0135 (11)	0.0177 (12)	0.0121 (12)	−0.0047 (9)	0.0085 (9)	0.0008 (9)
C11	0.0228 (12)	0.0200 (12)	0.0166 (13)	0.0004 (10)	0.0149 (10)	0.0027 (10)
C5	0.0192 (12)	0.0108 (11)	0.0182 (13)	0.0020 (9)	0.0092 (10)	0.0024 (9)
C12	0.0157 (12)	0.0218 (13)	0.0207 (14)	−0.0074 (10)	0.0065 (10)	0.0012 (10)
C16	0.0217 (12)	0.0160 (12)	0.0179 (13)	0.0024 (10)	0.0127 (10)	0.0053 (10)
C13	0.0143 (11)	0.0132 (11)	0.0133 (12)	−0.0032 (9)	0.0085 (9)	0.0034 (9)
C15	0.0240 (13)	0.0212 (13)	0.0166 (13)	−0.0010 (10)	0.0144 (11)	0.0019 (10)
C6	0.0173 (12)	0.0140 (11)	0.0200 (13)	0.0027 (9)	0.0120 (10)	0.0014 (10)
C14	0.0164 (12)	0.0215 (13)	0.0199 (13)	−0.0055 (10)	0.0063 (10)	0.0031 (10)

### 1,1'-(Ethane-1,2-diyl)bis(3-tert-butylimidazolium) dibromide dihydrate (est01043_0m) Geometric parameters (Å, º)

**Table:**

O2—H2C	0.8695	C10—H9B	0.9800
O2—H2D	0.8698	C10—H9C	0.9800
O1—H1C	0.8699	C2—H4	0.94 (3)
O1—H1D	0.8697	C8—H2A	0.9900
N1—C1	1.327 (3)	C8—H2B	0.9900
N1—C9	1.505 (3)	C11—H7A	0.9800
N1—C2	1.388 (3)	C11—H7B	0.9800
N2—C1	1.331 (3)	C11—H7C	0.9800
N2—C7	1.468 (3)	C5—C6	1.352 (3)
N2—C3	1.384 (3)	C5—H10	0.91 (3)
N3—C4	1.333 (3)	C12—H8A	0.9800
N3—C8	1.468 (3)	C12—H8B	0.9800
N3—C5	1.381 (3)	C12—H8C	0.9800
N4—C4	1.330 (3)	C16—H16A	0.9800
N4—C13	1.503 (3)	C16—H16B	0.9800
N4—C6	1.384 (3)	C16—H16C	0.9800
C1—H5	0.91 (2)	C16—C13	1.520 (3)
C9—C10	1.520 (3)	C13—C15	1.522 (3)
C9—C11	1.526 (3)	C13—C14	1.531 (3)
C9—C12	1.528 (3)	C15—H15A	0.9800
C7—H1A	0.9900	C15—H15B	0.9800
C7—H1B	0.9900	C15—H15C	0.9800
C7—C8	1.518 (3)	C6—H11	0.93 (3)
C4—H12	0.91 (2)	C14—H14A	0.9800
C3—C2	1.349 (3)	C14—H14B	0.9800
C3—H3	0.90 (3)	C14—H14C	0.9800
C10—H9A	0.9800		
			
H2C—O2—H2D	104.5	N3—C8—H2B	109.7
H1C—O1—H1D	109.5	C7—C8—H2A	109.7
C1—N1—C9	126.71 (19)	C7—C8—H2B	109.7
C1—N1—C2	108.21 (19)	H2A—C8—H2B	108.2
C2—N1—C9	125.02 (18)	C9—C11—H7A	109.5
C1—N2—C7	124.95 (19)	C9—C11—H7B	109.5
C1—N2—C3	108.54 (19)	C9—C11—H7C	109.5
C3—N2—C7	126.4 (2)	H7A—C11—H7B	109.5
C4—N3—C8	124.51 (19)	H7A—C11—H7C	109.5
C4—N3—C5	108.75 (19)	H7B—C11—H7C	109.5
C5—N3—C8	126.7 (2)	N3—C5—H10	118.4 (19)
C4—N4—C13	125.94 (19)	C6—C5—N3	106.7 (2)
C4—N4—C6	108.16 (19)	C6—C5—H10	134.9 (19)
C6—N4—C13	125.85 (19)	C9—C12—H8A	109.5
N1—C1—N2	109.02 (19)	C9—C12—H8B	109.5
N1—C1—H5	126.1 (16)	C9—C12—H8C	109.5
N2—C1—H5	124.9 (16)	H8A—C12—H8B	109.5
N1—C9—C10	108.60 (18)	H8A—C12—H8C	109.5
N1—C9—C11	107.87 (18)	H8B—C12—H8C	109.5
N1—C9—C12	107.13 (18)	H16A—C16—H16B	109.5
C10—C9—C11	110.2 (2)	H16A—C16—H16C	109.5
C10—C9—C12	110.8 (2)	H16B—C16—H16C	109.5
C11—C9—C12	112.13 (19)	C13—C16—H16A	109.5
N2—C7—H1A	109.7	C13—C16—H16B	109.5
N2—C7—H1B	109.7	C13—C16—H16C	109.5
N2—C7—C8	109.74 (18)	N4—C13—C16	108.52 (18)
H1A—C7—H1B	108.2	N4—C13—C15	108.11 (19)
C8—C7—H1A	109.7	N4—C13—C14	107.00 (19)
C8—C7—H1B	109.7	C16—C13—C15	110.4 (2)
N3—C4—H12	124.6 (15)	C16—C13—C14	110.6 (2)
N4—C4—N3	108.8 (2)	C15—C13—C14	112.0 (2)
N4—C4—H12	126.5 (15)	C13—C15—H15A	109.5
N2—C3—H3	120.5 (17)	C13—C15—H15B	109.5
C2—C3—N2	106.9 (2)	C13—C15—H15C	109.5
C2—C3—H3	132.5 (17)	H15A—C15—H15B	109.5
C9—C10—H9A	109.5	H15A—C15—H15C	109.5
C9—C10—H9B	109.5	H15B—C15—H15C	109.5
C9—C10—H9C	109.5	N4—C6—H11	121.8 (17)
H9A—C10—H9B	109.5	C5—C6—N4	107.6 (2)
H9A—C10—H9C	109.5	C5—C6—H11	130.7 (17)
H9B—C10—H9C	109.5	C13—C14—H14A	109.5
N1—C2—H4	120.4 (18)	C13—C14—H14B	109.5
C3—C2—N1	107.3 (2)	C13—C14—H14C	109.5
C3—C2—H4	132.3 (17)	H14A—C14—H14B	109.5
N3—C8—C7	109.68 (18)	H14A—C14—H14C	109.5
N3—C8—H2A	109.7	H14B—C14—H14C	109.5
			
N2—C7—C8—N3	−176.23 (18)	C4—N4—C6—C5	0.0 (3)
N2—C3—C2—N1	−0.2 (3)	C3—N2—C1—N1	−0.5 (3)
N3—C5—C6—N4	0.2 (3)	C3—N2—C7—C8	−86.8 (3)
C1—N1—C9—C10	2.3 (3)	C2—N1—C1—N2	0.4 (3)
C1—N1—C9—C11	121.7 (2)	C2—N1—C9—C10	179.3 (2)
C1—N1—C9—C12	−117.4 (2)	C2—N1—C9—C11	−61.3 (3)
C1—N1—C2—C3	−0.1 (3)	C2—N1—C9—C12	59.6 (3)
C1—N2—C7—C8	88.3 (3)	C8—N3—C4—N4	178.2 (2)
C1—N2—C3—C2	0.5 (3)	C8—N3—C5—C6	−178.1 (2)
C9—N1—C1—N2	177.7 (2)	C5—N3—C4—N4	0.4 (3)
C9—N1—C2—C3	−177.5 (2)	C5—N3—C8—C7	88.4 (3)
C7—N2—C1—N1	−176.37 (19)	C13—N4—C4—N3	−177.8 (2)
C7—N2—C3—C2	176.3 (2)	C13—N4—C6—C5	177.6 (2)
C4—N3—C8—C7	−88.9 (3)	C6—N4—C4—N3	−0.3 (3)
C4—N3—C5—C6	−0.4 (3)	C6—N4—C13—C16	176.4 (2)
C4—N4—C13—C16	−6.5 (3)	C6—N4—C13—C15	56.6 (3)
C4—N4—C13—C15	−126.3 (2)	C6—N4—C13—C14	−64.3 (3)
C4—N4—C13—C14	112.8 (2)		

### 1,1'-Methylenebis[3-(2,4,6-trimethylphenyl)imidazolium] dibromide dihydrate (at01019_0ma) Crystal data

**Table:**

C_25_H_30_N_4_^2+^·2Br^−^·2H_2_O	*D*_x_ = 1.416 Mg m^−^^3^
*M**_r_* = 582.38	Mo *K*α radiation, λ = 0.71073 Å
Orthorhombic, *P**c**c**n*	Cell parameters from 9959 reflections
*a* = 21.5695 (6) Å	θ = 2.6–39.4°
*b* = 28.3385 (6) Å	µ = 2.99 mm^−^^1^
*c* = 8.9401 (2) Å	*T* = 112 K
*V* = 5464.6 (2) Å^3^	Prism, clear colourless
*Z* = 8	0.4 × 0.3 × 0.25 mm
*F*(000) = 2384	

### 1,1'-Methylenebis[3-(2,4,6-trimethylphenyl)imidazolium] dibromide dihydrate (at01019_0ma) Data collection

**Table:**

Bruker Venture Kappa D diffractometer	5530 reflections with *I* > 2σ(*I*)
φ and ω scans	*R*_int_ = 0.035
Absorption correction: multi-scan (SADABS; Bruker, 2016)	θ_max_ = 27.1°, θ_min_ = 2.6°
*T*_min_ = 0.386, *T*_max_ = 0.748	*h* = −24→27
39085 measured reflections	*k* = −31→36
5954 independent reflections	*l* = −11→11

### 1,1'-Methylenebis[3-(2,4,6-trimethylphenyl)imidazolium] dibromide dihydrate (at01019_0ma) Refinement

**Table:**

Refinement on *F*^2^	Primary atom site location: iterative
Least-squares matrix: full	Hydrogen site location: mixed
*R*[*F*^2^ > 2σ(*F*^2^)] = 0.032	H atoms treated by a mixture of independent and constrained refinement
*wR*(*F*^2^) = 0.066	*w* = 1/[σ^2^(*F*_o_^2^) + (0.0149*P*)^2^ + 6.9269*P*] where *P* = (*F*_o_^2^ + 2*F*_c_^2^)/3
*S* = 1.19	(Δ/σ)_max_ = 0.002
5954 reflections	Δρ_max_ = 0.42 e Å^−^^3^
338 parameters	Δρ_min_ = −0.54 e Å^−^^3^
0 restraints	

### 1,1'-Methylenebis[3-(2,4,6-trimethylphenyl)imidazolium] dibromide dihydrate (at01019_0ma) Special details

**Table:**

Geometry. All esds (except the esd in the dihedral angle between two l.s. planes) are estimated using the full covariance matrix. The cell esds are taken into account individually in the estimation of esds in distances, angles and torsion angles; correlations between esds in cell parameters are only used when they are defined by crystal symmetry. An approximate (isotropic) treatment of cell esds is used for estimating esds involving l.s. planes.

### 1,1'-Methylenebis[3-(2,4,6-trimethylphenyl)imidazolium] dibromide dihydrate (at01019_0ma) Fractional atomic coordinates and isotropic or equivalent isotropic displacement parameters (Å^2^)

**Table:**

	*x*	*y*	*z*	*U*_iso_*/*U*_eq_	Occ. (<1)
Br1	0.32147 (2)	0.59206 (2)	0.51936 (2)	0.01792 (6)	
Br2	0.33241 (2)	0.42996 (2)	0.77256 (2)	0.01874 (6)	
N2	0.14868 (8)	0.40218 (6)	0.76624 (19)	0.0134 (3)	
N3	0.17753 (8)	0.54615 (6)	0.5774 (2)	0.0167 (4)	
N1	0.17956 (8)	0.47256 (6)	0.7130 (2)	0.0158 (4)	
N4	0.14048 (8)	0.61532 (6)	0.53485 (19)	0.0151 (3)	
C4	0.17963 (9)	0.42757 (7)	0.6667 (2)	0.0165 (4)	
H4	0.198554	0.415943	0.578146	0.020*	
C16	0.17313 (9)	0.59006 (7)	0.6302 (2)	0.0154 (4)	
H16	0.190588	0.601214	0.721117	0.018*	
C17	0.12153 (9)	0.66386 (7)	0.5581 (2)	0.0146 (4)	
C5	0.13900 (9)	0.35172 (7)	0.7556 (2)	0.0141 (4)	
O2A	0.4650 (10)	0.5643 (7)	0.601 (3)	0.037 (3)	0.47 (9)
H2AA	0.482702	0.563311	0.513609	0.055*	0.47 (9)
H2AB	0.427586	0.574062	0.582766	0.055*	0.47 (9)
C12	0.22444 (10)	0.34048 (8)	0.9479 (2)	0.0201 (4)	
H12A	0.256440	0.357156	0.890780	0.030*	
H12B	0.243324	0.314106	1.002226	0.030*	
H12C	0.205240	0.362270	1.019314	0.030*	
C14	0.14765 (13)	0.54398 (8)	0.4406 (3)	0.0275 (5)	
C18	0.07474 (9)	0.67224 (7)	0.6624 (2)	0.0150 (4)	
C22	0.15021 (10)	0.69938 (7)	0.4754 (2)	0.0179 (4)	
C6	0.17572 (9)	0.32189 (7)	0.8424 (2)	0.0153 (4)	
C24	0.04581 (10)	0.63259 (7)	0.7507 (2)	0.0190 (4)	
H24A	0.037277	0.605939	0.683916	0.028*	
H24B	0.006967	0.643518	0.795745	0.028*	
H24C	0.074401	0.622543	0.829693	0.028*	
C3	0.12848 (12)	0.43164 (8)	0.8801 (3)	0.0244 (5)	
C10	0.09279 (10)	0.33558 (7)	0.6589 (2)	0.0163 (4)	
C15	0.12434 (13)	0.58713 (8)	0.4143 (3)	0.0272 (5)	
C1	0.21637 (10)	0.50965 (7)	0.6432 (3)	0.0231 (5)	
H1A	0.243891	0.524043	0.719194	0.028*	
H1B	0.242843	0.495638	0.564401	0.028*	
C19	0.05623 (10)	0.71864 (7)	0.6822 (2)	0.0174 (4)	
H19	0.024420	0.725449	0.752452	0.021*	
C11	0.05222 (11)	0.36931 (8)	0.5741 (3)	0.0242 (5)	
H11A	0.036347	0.393487	0.642452	0.036*	
H11B	0.017393	0.352060	0.529724	0.036*	
H11C	0.076445	0.384406	0.494617	0.036*	
O1A	0.47220 (10)	0.47135 (8)	0.6838 (4)	0.0715 (9)	
H1AA	0.487824	0.450597	0.622561	0.107*	
H1AB	0.436751	0.459386	0.710574	0.107*	
C7	0.16563 (9)	0.27361 (7)	0.8269 (2)	0.0175 (4)	
H7	0.189642	0.252345	0.885177	0.021*	
C2	0.14808 (12)	0.47542 (8)	0.8472 (3)	0.0245 (5)	
C20	0.08292 (10)	0.75565 (7)	0.6021 (2)	0.0182 (4)	
C23	0.20108 (11)	0.68858 (8)	0.3644 (3)	0.0263 (5)	
H23A	0.236552	0.674719	0.416963	0.040*	
H23B	0.214017	0.717788	0.314740	0.040*	
H23C	0.185662	0.666212	0.289523	0.040*	
C8	0.12146 (10)	0.25546 (7)	0.7288 (2)	0.0184 (4)	
C9	0.08556 (10)	0.28686 (7)	0.6465 (2)	0.0179 (4)	
H9	0.055181	0.274754	0.579845	0.021*	
C13	0.11192 (11)	0.20288 (7)	0.7166 (3)	0.0243 (5)	
H13A	0.152068	0.186833	0.723425	0.036*	
H13B	0.092477	0.195470	0.620360	0.036*	
H13C	0.085012	0.192160	0.798149	0.036*	
C21	0.12978 (10)	0.74543 (7)	0.5009 (2)	0.0197 (4)	
H21	0.148577	0.770574	0.447203	0.024*	
C25	0.06032 (11)	0.80563 (7)	0.6251 (3)	0.0256 (5)	
H25A	0.020408	0.809802	0.574412	0.038*	
H25B	0.090640	0.827784	0.583474	0.038*	
H25C	0.055261	0.811696	0.732324	0.038*	
H2	0.1460 (13)	0.5020 (10)	0.892 (3)	0.031*	
H15	0.1018 (13)	0.5989 (9)	0.332 (3)	0.031*	
H3	0.1080 (13)	0.4191 (10)	0.963 (3)	0.031*	
H14	0.1479 (13)	0.5182 (10)	0.388 (3)	0.031*	
O2	0.4729 (8)	0.5614 (8)	0.556 (4)	0.043 (5)	0.53 (9)
H2A	0.438 (4)	0.569 (4)	0.542 (11)	0.064*	0.53 (9)
H2B	0.465 (4)	0.542 (3)	0.606 (11)	0.064*	0.53 (9)

### 1,1'-Methylenebis[3-(2,4,6-trimethylphenyl)imidazolium] dibromide dihydrate (at01019_0ma) Atomic displacement parameters (Å^2^)

**Table:**

	*U*^11^	*U*^22^	*U*^33^	*U*^12^	*U*^13^	*U*^23^
Br1	0.01793 (11)	0.01900 (10)	0.01682 (11)	−0.00262 (8)	0.00203 (8)	−0.00139 (8)
Br2	0.02215 (11)	0.01532 (10)	0.01876 (11)	−0.00184 (8)	0.00044 (8)	−0.00160 (8)
N2	0.0173 (8)	0.0108 (8)	0.0120 (8)	−0.0006 (6)	0.0010 (6)	−0.0006 (6)
N3	0.0177 (9)	0.0101 (8)	0.0222 (9)	0.0009 (6)	0.0016 (7)	0.0035 (7)
N1	0.0145 (8)	0.0116 (8)	0.0213 (9)	0.0005 (6)	0.0008 (7)	0.0025 (7)
N4	0.0207 (9)	0.0114 (8)	0.0133 (8)	0.0006 (6)	−0.0008 (7)	0.0006 (7)
C4	0.0157 (10)	0.0147 (9)	0.0190 (10)	0.0021 (8)	0.0023 (8)	0.0000 (8)
C16	0.0150 (10)	0.0125 (9)	0.0186 (10)	−0.0017 (7)	−0.0002 (8)	0.0012 (8)
C17	0.0179 (10)	0.0113 (9)	0.0147 (10)	0.0014 (7)	−0.0043 (8)	−0.0008 (8)
C5	0.0186 (10)	0.0101 (9)	0.0135 (10)	−0.0020 (7)	0.0038 (8)	0.0000 (7)
O2A	0.026 (5)	0.040 (4)	0.045 (6)	0.008 (4)	0.005 (4)	0.005 (5)
C12	0.0208 (11)	0.0196 (10)	0.0200 (11)	−0.0019 (8)	−0.0023 (9)	0.0016 (9)
C14	0.0443 (15)	0.0135 (10)	0.0248 (12)	0.0035 (10)	−0.0058 (11)	−0.0070 (9)
C18	0.0174 (10)	0.0128 (9)	0.0148 (10)	−0.0008 (7)	−0.0047 (8)	0.0020 (8)
C22	0.0198 (11)	0.0149 (10)	0.0192 (10)	−0.0008 (8)	−0.0009 (8)	0.0011 (8)
C6	0.0135 (10)	0.0168 (9)	0.0157 (10)	−0.0010 (7)	0.0034 (8)	0.0016 (8)
C24	0.0206 (11)	0.0157 (10)	0.0205 (11)	0.0016 (8)	0.0014 (9)	0.0045 (8)
C3	0.0376 (14)	0.0183 (11)	0.0174 (11)	0.0000 (9)	0.0088 (10)	−0.0005 (9)
C10	0.0177 (10)	0.0163 (10)	0.0150 (10)	−0.0026 (8)	0.0008 (8)	0.0027 (8)
C15	0.0446 (15)	0.0182 (11)	0.0188 (11)	0.0055 (10)	−0.0107 (11)	−0.0043 (9)
C1	0.0170 (11)	0.0122 (10)	0.0402 (14)	0.0011 (8)	0.0022 (10)	0.0103 (9)
C19	0.0182 (10)	0.0151 (10)	0.0188 (10)	0.0023 (8)	−0.0018 (8)	−0.0007 (8)
C11	0.0257 (12)	0.0202 (11)	0.0266 (12)	−0.0037 (9)	−0.0083 (10)	0.0042 (9)
O1A	0.0296 (12)	0.0545 (14)	0.130 (3)	0.0055 (10)	0.0180 (14)	0.0468 (16)
C7	0.0168 (10)	0.0147 (9)	0.0208 (10)	0.0018 (8)	0.0026 (8)	0.0041 (8)
C2	0.0364 (14)	0.0154 (10)	0.0216 (12)	0.0015 (9)	0.0050 (10)	−0.0040 (9)
C20	0.0212 (11)	0.0107 (9)	0.0227 (11)	0.0007 (8)	−0.0062 (9)	−0.0007 (8)
C23	0.0283 (13)	0.0216 (11)	0.0291 (13)	0.0004 (9)	0.0091 (10)	0.0042 (10)
C8	0.0202 (10)	0.0147 (10)	0.0201 (10)	−0.0028 (8)	0.0083 (8)	−0.0002 (8)
C9	0.0203 (11)	0.0170 (10)	0.0164 (10)	−0.0057 (8)	0.0002 (8)	−0.0013 (8)
C13	0.0267 (12)	0.0148 (10)	0.0313 (13)	−0.0033 (8)	0.0054 (10)	−0.0026 (9)
C21	0.0225 (11)	0.0125 (9)	0.0242 (11)	−0.0027 (8)	−0.0021 (9)	0.0036 (9)
C25	0.0283 (12)	0.0120 (10)	0.0366 (14)	0.0028 (9)	0.0002 (10)	−0.0008 (10)
O2	0.023 (4)	0.045 (5)	0.060 (13)	0.013 (3)	0.006 (5)	0.026 (7)

### 1,1'-Methylenebis[3-(2,4,6-trimethylphenyl)imidazolium] dibromide dihydrate (at01019_0ma) Geometric parameters (Å, º)

**Table:**

N2—C4	1.325 (3)	C3—C2	1.343 (3)
N2—C5	1.448 (2)	C3—H3	0.93 (3)
N2—C3	1.387 (3)	C10—C11	1.502 (3)
N3—C16	1.334 (3)	C10—C9	1.394 (3)
N3—C14	1.384 (3)	C15—H15	0.94 (3)
N3—C1	1.455 (3)	C1—H1A	0.9900
N1—C4	1.340 (3)	C1—H1B	0.9900
N1—C1	1.458 (3)	C19—H19	0.9500
N1—C2	1.380 (3)	C19—C20	1.394 (3)
N4—C16	1.317 (3)	C11—H11A	0.9800
N4—C17	1.450 (2)	C11—H11B	0.9800
N4—C15	1.386 (3)	C11—H11C	0.9800
C4—H4	0.9500	O1A—H1AA	0.8712
C16—H16	0.9500	O1A—H1AB	0.8701
C17—C18	1.394 (3)	C7—H7	0.9500
C17—C22	1.394 (3)	C7—C8	1.394 (3)
C5—C6	1.394 (3)	C2—H2	0.86 (3)
C5—C10	1.396 (3)	C20—C21	1.387 (3)
O2A—H2AA	0.8694	C20—C25	1.512 (3)
O2A—H2AB	0.8679	C23—H23A	0.9800
C12—H12A	0.9800	C23—H23B	0.9800
C12—H12B	0.9800	C23—H23C	0.9800
C12—H12C	0.9800	C8—C9	1.390 (3)
C12—C6	1.507 (3)	C8—C13	1.508 (3)
C14—C15	1.343 (3)	C9—H9	0.9500
C14—H14	0.87 (3)	C13—H13A	0.9800
C18—C24	1.508 (3)	C13—H13B	0.9800
C18—C19	1.386 (3)	C13—H13C	0.9800
C22—C23	1.511 (3)	C21—H21	0.9500
C22—C21	1.396 (3)	C25—H25A	0.9800
C6—C7	1.392 (3)	C25—H25B	0.9800
C24—H24A	0.9800	C25—H25C	0.9800
C24—H24B	0.9800	O2—H2A	0.79 (9)
C24—H24C	0.9800	O2—H2B	0.74 (11)
			
C4—N2—C5	124.39 (17)	C14—C15—N4	107.1 (2)
C4—N2—C3	108.93 (17)	C14—C15—H15	130.8 (17)
C3—N2—C5	126.68 (17)	N3—C1—N1	111.83 (17)
C16—N3—C14	108.72 (18)	N3—C1—H1A	109.3
C16—N3—C1	124.13 (19)	N3—C1—H1B	109.3
C14—N3—C1	126.41 (19)	N1—C1—H1A	109.3
C4—N1—C1	123.56 (19)	N1—C1—H1B	109.3
C4—N1—C2	108.94 (18)	H1A—C1—H1B	107.9
C2—N1—C1	126.71 (19)	C18—C19—H19	119.0
C16—N4—C17	124.98 (17)	C18—C19—C20	122.0 (2)
C16—N4—C15	108.91 (17)	C20—C19—H19	119.0
C15—N4—C17	126.00 (18)	C10—C11—H11A	109.5
N2—C4—N1	107.98 (18)	C10—C11—H11B	109.5
N2—C4—H4	126.0	C10—C11—H11C	109.5
N1—C4—H4	126.0	H11A—C11—H11B	109.5
N3—C16—H16	125.8	H11A—C11—H11C	109.5
N4—C16—N3	108.43 (19)	H11B—C11—H11C	109.5
N4—C16—H16	125.8	H1AA—O1A—H1AB	104.5
C18—C17—N4	117.50 (17)	C6—C7—H7	118.9
C22—C17—N4	118.93 (18)	C6—C7—C8	122.13 (19)
C22—C17—C18	123.56 (18)	C8—C7—H7	118.9
C6—C5—N2	118.71 (18)	N1—C2—H2	119.2 (19)
C6—C5—C10	123.44 (18)	C3—C2—N1	106.9 (2)
C10—C5—N2	117.85 (18)	C3—C2—H2	133.8 (19)
H2AA—O2A—H2AB	104.6	C19—C20—C25	120.1 (2)
H12A—C12—H12B	109.5	C21—C20—C19	118.61 (19)
H12A—C12—H12C	109.5	C21—C20—C25	121.3 (2)
H12B—C12—H12C	109.5	C22—C23—H23A	109.5
C6—C12—H12A	109.5	C22—C23—H23B	109.5
C6—C12—H12B	109.5	C22—C23—H23C	109.5
C6—C12—H12C	109.5	H23A—C23—H23B	109.5
N3—C14—H14	121.0 (18)	H23A—C23—H23C	109.5
C15—C14—N3	106.8 (2)	H23B—C23—H23C	109.5
C15—C14—H14	132.2 (19)	C7—C8—C13	120.2 (2)
C17—C18—C24	121.48 (18)	C9—C8—C7	118.53 (19)
C19—C18—C17	117.11 (19)	C9—C8—C13	121.2 (2)
C19—C18—C24	121.41 (19)	C10—C9—H9	119.0
C17—C22—C23	121.64 (19)	C8—C9—C10	122.0 (2)
C17—C22—C21	116.6 (2)	C8—C9—H9	119.0
C21—C22—C23	121.72 (19)	C8—C13—H13A	109.5
C5—C6—C12	122.16 (18)	C8—C13—H13B	109.5
C7—C6—C5	116.87 (19)	C8—C13—H13C	109.5
C7—C6—C12	120.96 (19)	H13A—C13—H13B	109.5
C18—C24—H24A	109.5	H13A—C13—H13C	109.5
C18—C24—H24B	109.5	H13B—C13—H13C	109.5
C18—C24—H24C	109.5	C22—C21—H21	118.9
H24A—C24—H24B	109.5	C20—C21—C22	122.1 (2)
H24A—C24—H24C	109.5	C20—C21—H21	118.9
H24B—C24—H24C	109.5	C20—C25—H25A	109.5
N2—C3—H3	120.2 (17)	C20—C25—H25B	109.5
C2—C3—N2	107.2 (2)	C20—C25—H25C	109.5
C2—C3—H3	132.4 (17)	H25A—C25—H25B	109.5
C5—C10—C11	121.33 (18)	H25A—C25—H25C	109.5
C9—C10—C5	116.98 (19)	H25B—C25—H25C	109.5
C9—C10—C11	121.68 (19)	H2A—O2—H2B	94 (10)
N4—C15—H15	122.0 (17)		
			
N2—C5—C6—C12	0.8 (3)	C18—C17—C22—C23	179.6 (2)
N2—C5—C6—C7	−178.95 (18)	C18—C17—C22—C21	−0.1 (3)
N2—C5—C10—C11	−2.9 (3)	C18—C19—C20—C21	−0.7 (3)
N2—C5—C10—C9	177.94 (18)	C18—C19—C20—C25	178.7 (2)
N2—C3—C2—N1	0.5 (3)	C22—C17—C18—C24	−179.1 (2)
N3—C14—C15—N4	0.3 (3)	C22—C17—C18—C19	0.4 (3)
N4—C17—C18—C24	1.4 (3)	C6—C5—C10—C11	176.7 (2)
N4—C17—C18—C19	−178.99 (18)	C6—C5—C10—C9	−2.5 (3)
N4—C17—C22—C23	−1.0 (3)	C6—C7—C8—C9	−1.5 (3)
N4—C17—C22—C21	179.30 (18)	C6—C7—C8—C13	−179.6 (2)
C4—N2—C5—C6	101.6 (2)	C24—C18—C19—C20	179.5 (2)
C4—N2—C5—C10	−78.8 (3)	C3—N2—C4—N1	−0.4 (2)
C4—N2—C3—C2	−0.1 (3)	C3—N2—C5—C6	−77.5 (3)
C4—N1—C1—N3	116.6 (2)	C3—N2—C5—C10	102.1 (3)
C4—N1—C2—C3	−0.8 (3)	C10—C5—C6—C12	−178.8 (2)
C16—N3—C14—C15	−1.2 (3)	C10—C5—C6—C7	1.5 (3)
C16—N3—C1—N1	110.9 (2)	C15—N4—C16—N3	−1.4 (2)
C16—N4—C17—C18	−72.6 (3)	C15—N4—C17—C18	103.3 (3)
C16—N4—C17—C22	108.0 (2)	C15—N4—C17—C22	−76.2 (3)
C16—N4—C15—C14	0.7 (3)	C1—N3—C16—N4	172.30 (18)
C17—N4—C16—N3	175.03 (18)	C1—N3—C14—C15	−171.6 (2)
C17—N4—C15—C14	−175.7 (2)	C1—N1—C4—N2	171.14 (19)
C17—C18—C19—C20	0.0 (3)	C1—N1—C2—C3	−170.8 (2)
C17—C22—C21—C20	−0.6 (3)	C19—C20—C21—C22	1.0 (3)
C5—N2—C4—N1	−179.63 (18)	C11—C10—C9—C8	−177.7 (2)
C5—N2—C3—C2	179.1 (2)	C7—C8—C9—C10	0.4 (3)
C5—C6—C7—C8	0.6 (3)	C2—N1—C4—N2	0.7 (2)
C5—C10—C9—C8	1.5 (3)	C2—N1—C1—N3	−74.7 (3)
C12—C6—C7—C8	−179.16 (19)	C23—C22—C21—C20	179.7 (2)
C14—N3—C16—N4	1.6 (2)	C13—C8—C9—C10	178.5 (2)
C14—N3—C1—N1	−80.1 (3)	C25—C20—C21—C22	−178.4 (2)

### 1,1'-(Ethane-1,2-diyl)bis[3-(2,4,6-trimethylphenyl)imidazolium] dibromide tetrahydrate (est01041d_0ma) Crystal data

**Table:**

C_26_H_32_N_4_^2+^·2Br^−^·4H_2_O	*F*(000) = 652
*M**_r_* = 632.42	*D*_x_ = 1.461 Mg m^−^^3^
Monoclinic, *P*2_1_/*c*	Mo *K*α radiation, λ = 0.71073 Å
*a* = 12.4230 (3) Å	Cell parameters from 9565 reflections
*b* = 13.1447 (3) Å	θ = 2.3–44.6°
*c* = 9.2780 (2) Å	µ = 2.86 mm^−^^1^
β = 108.379 (1)°	*T* = 100 K
*V* = 1437.78 (6) Å^3^	Prism, clear colourless
*Z* = 2	0.15 × 0.15 × 0.05 mm

### 1,1'-(Ethane-1,2-diyl)bis[3-(2,4,6-trimethylphenyl)imidazolium] dibromide tetrahydrate (est01041d_0ma) Data collection

**Table:**

Bruker Venture D8 Kappa diffractometer	3028 reflections with *I* > 2σ(*I*)
φ and ω scans	*R*_int_ = 0.025
Absorption correction: multi-scan (SADABS; Bruker, 2016)	θ_max_ = 27.1°, θ_min_ = 2.8°
*T*_min_ = 0.544, *T*_max_ = 0.750	*h* = −15→15
28199 measured reflections	*k* = −16→16
3165 independent reflections	*l* = −11→11

### 1,1'-(Ethane-1,2-diyl)bis[3-(2,4,6-trimethylphenyl)imidazolium] dibromide tetrahydrate (est01041d_0ma) Refinement

**Table:**

Refinement on *F*^2^	Primary atom site location: dual
Least-squares matrix: full	Hydrogen site location: mixed
*R*[*F*^2^ > 2σ(*F*^2^)] = 0.018	H atoms treated by a mixture of independent and constrained refinement
*wR*(*F*^2^) = 0.044	*w* = 1/[σ^2^(*F*_o_^2^) + (0.0151*P*)^2^ + 0.8539*P*] where *P* = (*F*_o_^2^ + 2*F*_c_^2^)/3
*S* = 1.10	(Δ/σ)_max_ < 0.001
3165 reflections	Δρ_max_ = 0.33 e Å^−^^3^
194 parameters	Δρ_min_ = −0.29 e Å^−^^3^
0 restraints	

### 1,1'-(Ethane-1,2-diyl)bis[3-(2,4,6-trimethylphenyl)imidazolium] dibromide tetrahydrate (est01041d_0ma) Special details

**Table:**

Geometry. All esds (except the esd in the dihedral angle between two l.s. planes) are estimated using the full covariance matrix. The cell esds are taken into account individually in the estimation of esds in distances, angles and torsion angles; correlations between esds in cell parameters are only used when they are defined by crystal symmetry. An approximate (isotropic) treatment of cell esds is used for estimating esds involving l.s. planes.

### 1,1'-(Ethane-1,2-diyl)bis[3-(2,4,6-trimethylphenyl)imidazolium] dibromide tetrahydrate (est01041d_0ma) Fractional atomic coordinates and isotropic or equivalent isotropic displacement parameters (Å^2^)

**Table:**

	*x*	*y*	*z*	*U*_iso_*/*U*_eq_	
Br01	−0.19328 (2)	0.01828 (2)	0.22033 (2)	0.01558 (5)	
O1	−0.07000 (10)	0.23627 (9)	0.37843 (13)	0.0268 (2)	
H1D	−0.1073 (18)	0.1876 (18)	0.340 (2)	0.036 (5)*	
H1E	−0.0360 (18)	0.2528 (17)	0.326 (3)	0.039 (6)*	
N1	0.28176 (8)	0.55577 (8)	0.81318 (11)	0.0117 (2)	
N2	0.12491 (9)	0.48768 (8)	0.67428 (12)	0.0123 (2)	
O2	0.04964 (10)	0.29104 (10)	0.18398 (14)	0.0276 (2)	
H2A	0.0823 (18)	0.3420 (18)	0.200 (2)	0.036 (6)*	
H2B	0.016 (2)	0.2902 (19)	0.096 (3)	0.051 (7)*	
C12	0.71245 (12)	0.75891 (11)	0.97675 (18)	0.0250 (3)	
H1A	0.736386	0.766617	0.886291	0.037*	
H1B	0.768542	0.718094	1.052694	0.037*	
H1C	0.706272	0.826152	1.019087	0.037*	
C6	0.59853 (11)	0.70628 (10)	0.93381 (15)	0.0166 (3)	
C10	0.51141 (11)	0.74146 (10)	0.98445 (14)	0.0166 (3)	
H3	0.523920	0.800361	1.046905	0.020*	
C8	0.40605 (11)	0.69326 (10)	0.94673 (14)	0.0148 (2)	
C9	0.39084 (10)	0.60639 (9)	0.85679 (13)	0.0117 (2)	
C1	0.21894 (10)	0.53490 (9)	0.67103 (14)	0.0126 (2)	
H6	0.2362 (13)	0.5503 (13)	0.5832 (19)	0.015 (4)*	
C7	0.03385 (10)	0.45390 (10)	0.53959 (14)	0.0132 (2)	
H7A	−0.016689	0.406014	0.569818	0.016*	
H7B	0.066579	0.418089	0.469255	0.016*	
C4	0.47678 (10)	0.56704 (10)	0.80436 (13)	0.0128 (2)	
C5	0.57920 (10)	0.61949 (10)	0.84337 (14)	0.0147 (2)	
H9	0.637928	0.595239	0.807044	0.018*	
C13	0.46334 (11)	0.47141 (10)	0.71025 (16)	0.0183 (3)	
H10A	0.418593	0.486447	0.604922	0.027*	
H10B	0.424390	0.419506	0.751255	0.027*	
H10C	0.538291	0.446186	0.713460	0.027*	
C11	0.31461 (12)	0.73712 (11)	1.00412 (16)	0.0225 (3)	
H11A	0.240263	0.726481	0.927945	0.034*	
H11B	0.327739	0.810168	1.022662	0.034*	
H11C	0.316568	0.703141	1.098930	0.034*	
C3	0.12649 (11)	0.47941 (11)	0.82365 (15)	0.0166 (3)	
H12	0.0660 (15)	0.4502 (14)	0.8469 (19)	0.023 (4)*	
C2	0.22470 (11)	0.52150 (11)	0.91051 (15)	0.0162 (3)	
H13	0.2517 (15)	0.5293 (13)	1.016 (2)	0.022 (4)*	

### 1,1'-(Ethane-1,2-diyl)bis[3-(2,4,6-trimethylphenyl)imidazolium] dibromide tetrahydrate (est01041d_0ma) Atomic displacement parameters (Å^2^)

**Table:**

	*U*^11^	*U*^22^	*U*^33^	*U*^12^	*U*^13^	*U*^23^
Br01	0.01802 (7)	0.01898 (8)	0.01231 (7)	−0.00240 (5)	0.00847 (5)	−0.00121 (5)
O1	0.0320 (6)	0.0288 (6)	0.0207 (5)	−0.0124 (5)	0.0100 (5)	−0.0066 (5)
N1	0.0124 (5)	0.0137 (5)	0.0093 (5)	0.0009 (4)	0.0037 (4)	0.0012 (4)
N2	0.0122 (5)	0.0141 (5)	0.0108 (5)	−0.0002 (4)	0.0039 (4)	0.0020 (4)
O2	0.0332 (6)	0.0284 (6)	0.0201 (6)	−0.0086 (5)	0.0068 (5)	0.0010 (5)
C12	0.0165 (6)	0.0195 (7)	0.0338 (8)	−0.0033 (5)	0.0007 (6)	−0.0009 (6)
C6	0.0159 (6)	0.0141 (6)	0.0158 (6)	−0.0007 (5)	−0.0005 (5)	0.0031 (5)
C10	0.0206 (6)	0.0125 (6)	0.0133 (6)	0.0006 (5)	0.0003 (5)	−0.0027 (5)
C8	0.0168 (6)	0.0150 (6)	0.0112 (5)	0.0045 (5)	0.0024 (4)	0.0008 (5)
C9	0.0116 (5)	0.0134 (6)	0.0087 (5)	−0.0002 (4)	0.0012 (4)	0.0018 (4)
C1	0.0141 (6)	0.0132 (6)	0.0109 (6)	−0.0006 (4)	0.0046 (5)	0.0007 (4)
C7	0.0125 (5)	0.0136 (6)	0.0120 (5)	−0.0024 (5)	0.0021 (4)	−0.0005 (5)
C4	0.0153 (6)	0.0127 (6)	0.0094 (5)	0.0015 (5)	0.0027 (4)	0.0008 (5)
C5	0.0136 (5)	0.0161 (6)	0.0140 (6)	0.0018 (5)	0.0036 (4)	0.0020 (5)
C13	0.0178 (6)	0.0179 (7)	0.0200 (6)	−0.0004 (5)	0.0070 (5)	−0.0070 (5)
C11	0.0222 (7)	0.0238 (7)	0.0219 (7)	0.0057 (5)	0.0076 (5)	−0.0074 (6)
C3	0.0155 (6)	0.0228 (7)	0.0127 (6)	0.0005 (5)	0.0064 (5)	0.0050 (5)
C2	0.0164 (6)	0.0224 (7)	0.0109 (6)	0.0025 (5)	0.0059 (5)	0.0036 (5)

### 1,1'-(Ethane-1,2-diyl)bis[3-(2,4,6-trimethylphenyl)imidazolium] dibromide tetrahydrate (est01041d_0ma) Geometric parameters (Å, º)

**Table:**

O1—H1D	0.80 (2)	C8—C11	1.5126 (17)
O1—H1E	0.77 (2)	C9—C4	1.4042 (17)
N1—C9	1.4481 (15)	C1—H6	0.928 (17)
N1—C1	1.3322 (16)	C7—C7^i^	1.525 (2)
N1—C2	1.3872 (16)	C7—H7A	0.9900
N2—C1	1.3314 (16)	C7—H7B	0.9900
N2—C7	1.4653 (16)	C4—C5	1.3909 (17)
N2—C3	1.3841 (16)	C4—C13	1.5095 (17)
O2—H2A	0.77 (2)	C5—H9	0.9500
O2—H2B	0.79 (3)	C13—H10A	0.9800
C12—H1A	0.9800	C13—H10B	0.9800
C12—H1B	0.9800	C13—H10C	0.9800
C12—H1C	0.9800	C11—H11A	0.9800
C12—C6	1.5114 (18)	C11—H11B	0.9800
C6—C10	1.3880 (19)	C11—H11C	0.9800
C6—C5	1.3915 (18)	C3—H12	0.928 (18)
C10—H3	0.9500	C3—C2	1.3506 (19)
C10—C8	1.3959 (18)	C2—H13	0.932 (19)
C8—C9	1.3915 (18)		
			
H1D—O1—H1E	107 (2)	N2—C7—H7A	109.8
C1—N1—C9	125.15 (10)	N2—C7—H7B	109.8
C1—N1—C2	108.51 (11)	C7^i^—C7—H7A	109.8
C2—N1—C9	126.34 (10)	C7^i^—C7—H7B	109.8
C1—N2—C7	124.67 (11)	H7A—C7—H7B	108.3
C1—N2—C3	108.89 (11)	C9—C4—C13	123.34 (11)
C3—N2—C7	126.41 (11)	C5—C4—C9	117.41 (11)
H2A—O2—H2B	106 (2)	C5—C4—C13	119.25 (11)
H1A—C12—H1B	109.5	C6—C5—H9	118.9
H1A—C12—H1C	109.5	C4—C5—C6	122.21 (12)
H1B—C12—H1C	109.5	C4—C5—H9	118.9
C6—C12—H1A	109.5	C4—C13—H10A	109.5
C6—C12—H1B	109.5	C4—C13—H10B	109.5
C6—C12—H1C	109.5	C4—C13—H10C	109.5
C10—C6—C12	121.57 (12)	H10A—C13—H10B	109.5
C10—C6—C5	118.20 (12)	H10A—C13—H10C	109.5
C5—C6—C12	120.23 (12)	H10B—C13—H10C	109.5
C6—C10—H3	118.9	C8—C11—H11A	109.5
C6—C10—C8	122.24 (12)	C8—C11—H11B	109.5
C8—C10—H3	118.9	C8—C11—H11C	109.5
C10—C8—C11	119.19 (12)	H11A—C11—H11B	109.5
C9—C8—C10	117.53 (12)	H11A—C11—H11C	109.5
C9—C8—C11	123.28 (12)	H11B—C11—H11C	109.5
C8—C9—N1	118.87 (11)	N2—C3—H12	120.6 (11)
C8—C9—C4	122.39 (11)	C2—C3—N2	106.87 (11)
C4—C9—N1	118.73 (11)	C2—C3—H12	132.6 (11)
N1—C1—H6	126.8 (10)	N1—C2—H13	123.8 (11)
N2—C1—N1	108.54 (11)	C3—C2—N1	107.19 (11)
N2—C1—H6	124.6 (10)	C3—C2—H13	129.0 (11)
N2—C7—C7^i^	109.28 (13)		
			
N1—C9—C4—C5	177.64 (10)	C1—N1—C9—C4	−53.80 (17)
N1—C9—C4—C13	−2.91 (18)	C1—N1—C2—C3	0.17 (15)
N2—C3—C2—N1	0.44 (15)	C1—N2—C7—C7^i^	−73.94 (17)
C12—C6—C10—C8	−179.51 (12)	C1—N2—C3—C2	−0.90 (15)
C12—C6—C5—C4	178.27 (12)	C7—N2—C1—N1	179.04 (11)
C6—C10—C8—C9	0.85 (19)	C7—N2—C3—C2	−178.88 (12)
C6—C10—C8—C11	−179.00 (12)	C5—C6—C10—C8	−0.48 (19)
C10—C6—C5—C4	−0.78 (19)	C13—C4—C5—C6	−177.92 (12)
C10—C8—C9—N1	−178.79 (11)	C11—C8—C9—N1	1.04 (18)
C10—C8—C9—C4	0.00 (18)	C11—C8—C9—C4	179.84 (12)
C8—C9—C4—C5	−1.16 (18)	C3—N2—C1—N1	1.01 (14)
C8—C9—C4—C13	178.30 (12)	C3—N2—C7—C7^i^	103.73 (16)
C9—N1—C1—N2	179.64 (11)	C2—N1—C9—C8	−54.53 (17)
C9—N1—C2—C3	179.79 (12)	C2—N1—C9—C4	126.64 (13)
C9—C4—C5—C6	1.56 (18)	C2—N1—C1—N2	−0.73 (14)
C1—N1—C9—C8	125.04 (13)		

Symmetry code: (i) −*x*, −*y*+1, −*z*+1.
